# Supplementary material for: Robustly measuring multimorbidity using disparate linked datasets
Source: Commun Med (Lond). 2025 Jul 8;5:283. doi: 10.1038/s43856-025-00995-4 (PMC12238475; doi:10.1038/s43856-025-00995-4)
Supplement: Supplementary file 2 — Supplementary information [file 43856_2025_995_MOESM2_ESM.pdf]

Supplementary Information

Robustly measuring multimorbidity using disparate linked datasets

Regina Prigge[1*], Kelly J Fleetwood[1], Caroline A Jackson[1], Stewart W Mercer[1], Paul AT Kelly[2], Cathie Sudlow[1,3], John D Norrie[1], Daniel R Morales[4,5], Daniel J Smith[6], Bruce Guthrie[7]

1 Usher Institute, University of Edinburgh, Edinburgh, United Kingdom

2 Public member of study advisory board, Edinburgh, United Kingdom

3 British Heart Foundation Data Science Centre, Health Data Research UK, London, United Kingdom

4 Division of Population Health and Genomics, University of Dundee, Dundee, United Kingdom

5 Department of Public Health, University of Southern Denmark, Odense, Denmark

6 Centre for Clinical Brain Sciences, University of Edinburgh, Edinburgh, United Kingdom

7 Advanced Care Research Centre, Usher Institute, University of Edinburgh, Edinburgh, United Kingdom

* Corresponding author

Regina Prigge, Usher Institute, University of Edinburgh, BioQuarter - Gate, Usher Building, The University of Edinburgh, 5-7, 3 Little France Rd, Edinburgh EH16 4UX, UK, regina.prigge@ed.ac.uk

Table of contents

Supplementary Table 1. Availability of data ... 3

Supplementary Methods 1. Description of the process of choosing long-term conditions to include in the study 4

Supplementary Methods 2. Identification of conditions in primary care records ... 5

Supplementary Table 2. Overview of modifications to CALIBER definitions ... 7

Supplementary Table 3. Description of additional rules ... 8

Supplementary Fig. 3. Flow diagram of sample selection ... 9

Supplementary Fig. 4. Proportional Venn diagrams of concordance between data sources for all conditions by body system (using data from all three data sources) ... 10

Supplementary Fig. 5. Tornado plot of concordance between primary care and UK Biobank records for all conditions by body system ... 14

Supplementary Fig. 6. Tornado plot of concordance between primary care and hospital records for all conditions by body system. ... 15

Supplementary Fig. 7. Tornado plot of concordance between UK Biobank records and hospital records for all conditions by body system ... 16

Supplementary Fig. 8. Prevalence of multiple long-term health conditions by age, sex, ethnicity and deprivation for MLTC 3+ and MLTC 3+ from 3+ ... 17

Supplementary Table 4. Baseline characteristics of eligible participants for the whole population and for subsets of the population meeting each of the four MLTC definitions (MLTC ascertained using primary care records alone) ... 18

Supplementary Table 5. Baseline characteristics of eligible participants for the whole population and for subsets of the population meeting each of the four MLTC definitions (MLTC ascertained using UKB records alone) .. 19

Supplementary Table 6. Baseline characteristics of eligible participants for the whole population and for subsets of the population meeting each of the four MLTC definitions (MLTC ascertained using hospital records alone) ... 20

Supplementary References ... 21

Supplementary Table 1. Availability of data

Data source	Region	Data complete	Summary
Hospital admission	Scotland	Earliest record: Jan 1981<br>Complete to: 31 Aug 2022	For each participant: Allow for an 8-year look-back from the assessment centre date, and include records up to and including their baseline assessment date
	Wales	Earliest record: Jan 1998*<br>Complete to: 31 May 2022	
	England	Earliest record: Apr 1997<br>Complete to: 31 Oct 2022	
Cancer registry	Scotland	Earliest record: 1957<br>Complete to: 30 Nov 2021	For each participant: Allow for an 8-year look-back from the assessment centre date, and include records up to and including their baseline assessment date
	Wales	Earliest record: 1971<br>Complete to: 31 Dec 2016	
	England	Earliest record: 1971<br>Complete to: 31 Dec 2020	
Primary care	Scotland	Earliest record[1]: Aug 1937<br>Complete to: 31 Mar 2017	Assume that all key conditions have been captured for participants with a continuous GP record of at least a year at the time of baseline assessment[1]. For each participant, use all primary care records up to and including the date of their baseline assessment date
	Wales	Earliest record[#]: May 1940<br>Compete to: 31 Aug 2017	
	England (TPP)	Earliest record[1]: Dec 1937<br>Complete to: 31 May 2016	

Table legend:

* At time of publication, the UK Biobank website (https://biobank.ndph.ox.ac.uk/showcase/exinfo.cgi?src=Data_providers_and_dates) gives the start date for Wales as 1991, but data records aren't consistently available until 1 Jan 1998

\# Records prior to each participant's date of birth were excluded.

Supplementary Methods 1. Description of the process of choosing long-term conditions to include in the study

Two primary care physicians with a research focus on multimorbidity (BG, SM) and one psychiatrist specialising in bipolar disorder (DS) selected LTCs to include from a set of 308 conditions[2], with discussion in case of disagreement. Clinicians were asked to select conditions that were: 1) A long-term physical or mental health condition; and 2) Of relevance to middle-aged adults (the recruited population in UKB). After reaching clinical consensus, 154 conditions were selected (see "Sub-conditions" column in Supplementary Table 2). However, some conditions had to be grouped to avoid double-counting of the same underlying condition (for example, 'unstable angina', 'stable angina', 'myocardial infarction', and 'coronary heart disease unspecified' were all included in a single condition - 'coronary heart disease') in longitudinal analyses that were planned as part of the same project co-funded by the Medical Research Council and the National Institute for Health Research through grant number MC/S028013. We subsequently added two additional conditions (post-traumatic stress disorder and Addison's disease) following a Delphi consensus study published during our study that recommended conditions that should always or usually be included in research on MLTCs[3].

Supplementary Methods 2. Identification of conditions in primary care records

For each condition and coding system, we applied a two-step process of systematically identifying existing code lists and creating new code lists for conditions with no published code list. Where possible, we used existing and validated code-lists created by researchers using the CALIBER platform,[2,4] which are based on Read v2 coded primary care data, ICD-10 coded hospital data and OPCS-4 coded operations and procedures data. However, since the primary care data released by UK Biobank in September 2019 only included the first five digits of Read v2 codes, we had to convert all existing 7-digit Read version 2 code-lists to 5-digit Read v2 code-lists and account for resulting inconsistencies (Fig A, left column). In addition, the clinical review of code-lists led to a modification of some CALIBER definitions (Supplementary Appendix 6). For example, whilst the CALIBER definition of deafness was broad, we have used a narrower definition of deafness, including: participants with bilateral moderate or severe deafness and participants who use hearing aids. Where there was no CALIBER code-list (e.g., Addison's Disease), we created new Read v2 and ICD-10 code lists (Fig A, right column).

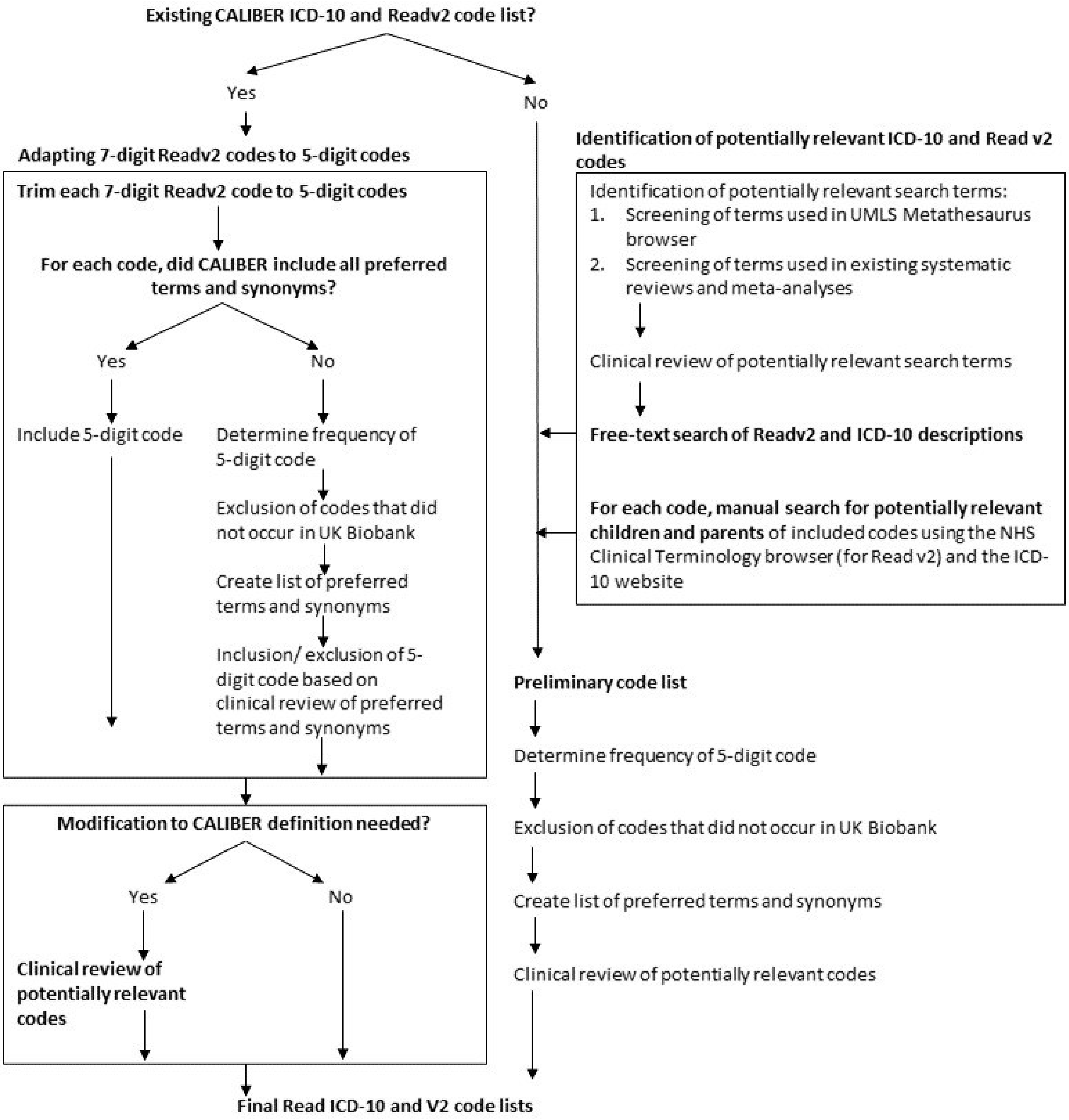

Supplementary Fig. 1: Development of Read v2 and ICD-10 code lists

We then applied a two-step process of systematically identifying existing CTV3 code-lists and creating new CTV3 code-lists for conditions with no published code-list (Fig B). For each condition, we searched for matching CTV3 code-lists in the OpenSafely repository,[5] on the UK Biobank website,[6] by

contacting topic experts, and through a review of existing literature. For each condition with at least one existing CTV3 code-list, we created a combined code-list containing all codes that were included in one or more existing code-list and any additional codes identified through mapping relevant Read v2 codes to potentially relevant CTV3 codes. For each condition with an existing Read v2 code-list but no existing CTV3 code-list, we developed new CTV3 code-lists. A clinician (BG) then selected clinically relevant codes for inclusion in our project.

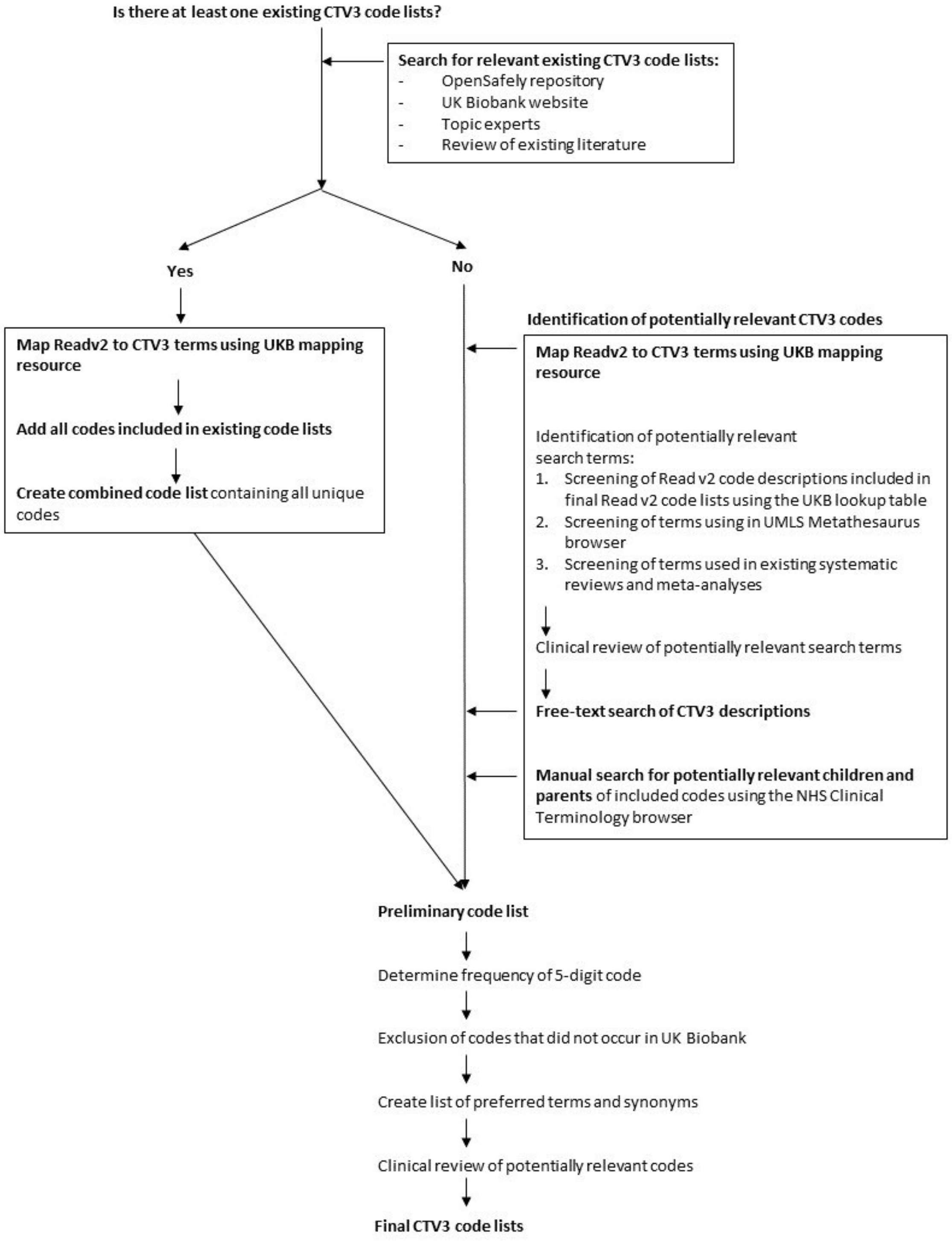

Supplementary Fig. 2: Development of CTV3 code lists

Supplementary Table 2. Overview of modifications to CALIBER definitions

Condition	Modification
Depression	For depression, we used a code list developed by Davidson and colleagues who categorised codes in their code list into five broad categories (Admin, Diagnosis, Drug, Symptom/Sign and Score/Screen) (Davidson E, Unversity of Edinburgh, personal communication). We included a subset of the Diagnosis codes that were relevant to our project.
Visual impairment and blindness	Kuan and colleagues used a broad definition of blindness including Read codes for loss of vision and Read codes for blindness in only one eye.[2] For this project, we have used a narrower definition of blindness, including only participants including only participants with at least moderate visual impairment in the better seeing eye.
Deafness	Kuan and colleagues defined 'hearing loss' using a broad definition of deafness. For this project, we have used a narrower definition of deafness, including:<br>1. participants with moderate or severe bilateral deafness<br>and<br>2. participants who use hearing aids.[2]
Diabetes	Kuan and colleagues used a modified CALIBER algorithm to differentiate between type 1 diabetes, type 2 diabetes and diabetes of other or uncertain type.[2] This algorithm requires two primary care code lists: one that specifically defines diabetes diagnosis codes and another that defines a wider set of diabetes codes. For diabetes, we only included codes that specifically indicated diabetes. We excluded codes where diabetes was mentioned in relation to another condition.
Epilepsy	During the identification of the history, administrative and procedure/treatment codes, we reviewed the codes in this list and decided to exclude infantile spasms from our definition of epilepsy.
Hypertension	During the identification of the history, administrative and procedure/treatment codes, we reviewed the codes in this list and decided to exclude borderline hypertension and treatment with antihypertensives from our definition of hypertension.
Chronic viral hepatitis	For chronic viral hepatitis we excluded laboratory procedures that could potentially return negative results. It was not possible evaluate each result against a cut-off because the laboratory results were often missing from the primary care dataset.
Alcoholic liver disease	Kuan and colleagues included alcoholic fatty liver in two codelists: Alcoholic liver disease and Fatty liver.[2] For this project, we excluded fatty liver Read codes from all codelists other than the fatty liver codelist.
Rheumatoid arthritis	During the development of the Read version 3 code list for rheumatoid arthritis, we also reviewed the codes included in Kuan et al.'s Read version 2 code list for this condition and excluded one code.
Hypo- or hyperthyroidism	During the development of the Read version 3 code list for hypo or hyperthyroidism, we also reviewed the codes included in Kuan et al.'s Read version 2 code list for this condition and excluded one code.
Tuberculosis	During the identification of the history, administrative and procedure/treatment codes, we reviewed the codes in this list and decided to exclude two codes from our definition of tuberculosis.
Alcohol problems	During the identification of the history, administrative and procedure/treatment codes, we reviewed the codes in this list and decided to exclude one code from our definition of alcohol problems.
Other psychoactive substance misuse	During the identification of the history, administrative and procedure/treatment codes, we reviewed the codes in this list and decided to exclude one code (adverse reaction to buprenorphine) from our definition of other psychoactive substance misuse.
Primary malignancy - other organs	Kuan and colleagues used a rule to identify 'primary malignancy - other organs'.[2] The rule used a combination of codes for 'primary malignancy - other organs' and codes for other malignancies. In this project, most primary malignancies were used to define a single category of 'solid organ malignancies'. We also defined separate categories for 'haematological malignancies' and 'non-melanoma skin malignancies'. We adapted Kuan et al.'s approach to defining 'primary malignancy - other organs' as follows:<br>• we reviewed the codes included in the 'primary malignancy - other organs' spreadsheet and identified codes specific to haematological malignancies and skin malignancies<br>• we moved the eight identified haematological malignancy codes to a new 'haematological malignancy - other' code list. This code list is included in the 'haematological malignancies' condition<br>• the two identified skin malignancy codes could not be assigned to an existing skin malignancy code list because the two codes were general codes (excision malignant skin tumour; melanoma and other malignant neoplasms of skin) and not specific to either 'non-melananoma skin malignanices' or 'malignant melanoma' (these individual conditions belong to separate lumped conditions). We decided to completely exclude these codes from our code lists because participants with one of these codes almost always had a specific 'non-melanoma skin malignancy' or 'malignant melanoma' code

Supplementary Table 3. Description of additional rules

Condition	Rule
Chronic kidney disease (sub-condition of chronic renal disease)	We combined codes for chronic kidney disease (CKD) with serum creatinine codes and serum creatinine measurements from the UK Biobank baseline assessment, as follows:<br><br>If the participant had a UK Biobank baseline assessment serum creatinine measurement, then<br>• We excluded other records (e.g. hospital records) of CKD up to and including the date of the baseline assessment<br>• We defined the participant to have CKD from the date of the baseline assessment if they had an eGFR of less than 60 ml per minute per 1.73 m2. We calculated eGFR from serum creatinine measurements using the CKD-EPI Creatinine Equation (2021).[7]<br><br>If the participant did not have a baseline assessment serum creatinine measurement, then we defined CKD if<br>• the person had a code for CKD, or<br>• if the person had two eGFR values in their primary care record of less than 60 ml per minute per 1.73 m2 within 90 days (in which case we took the date of the second value as the date of diagnosis). We calculated eGFR from serum creatinine measurements as above, excluding serum creatinine values less than 10 or greater than 2000. If there were multiple serum creatinine measurements on a single day, we used their mean value instead of the individual values. Our code list repository includes Read V2 and CTV3 code lists for serum creatinine.
Stroke	We excluded records of stroke not otherwise specified, if there was a subarachnoid haemorrhage record within the previous 28 days.
Diabetes	Participants can only have one type of diabetes. For people with any diabetes code (a code in the type 1, type 2 or diabetes not otherwise specified code lists), we identified diabetes type (type 1, type 2 or diabetes not otherwise specified) by applying a modified version of the algorithm used by Kuan and colleagues (see below).[2] We defined diabetes date of diagnosis as the earliest date of any diabetes code.<br><br>The algorithm prioritises type 1 or type 2 diabetes diagnosis codes (e.g. type 1 diabetes mellitus) from primary care data, over other codes from primary care data (e.g. dietary advice for type 1 diabetes) and codes from all other sources. The Read v2 and CTV3 code lists for type 1 and type 2 diabetes each include a Diagnosis column which defines whether each code is a diagnosis code (Diagnosis = Y) or not (Diagnosis = N).<br><br>Our modified algorithm is described below:<br>• If there is at least one primary care diagnosis code for type 2 diabetes and no primary care diagnosis code for type 1 diabetes, then classify the person as type 2 diabetes<br>• If there is at least one primary care diagnosis code for type 1 diabetes and no primary care diagnosis code for type 2 diabetes, then classify the person as type 1 diabetes<br>• Else if there are no primary care diagnosis codes, or the primary care diagnosis codes are inconsistent<br>o If there is at least one record for type 2 diabetes and no record for type 1 diabetes then classify the person as type 2 diabetes<br>o If there is at least one record for type 1 diabetes and no record for type 2 diabetes then classify the person as type 1 diabetes<br>• Else classify the person as diabetes not otherwise specified
Sex-specific conditions	The following conditions were treated as sex-specific:<br>Men only:<br>- Hyperplasia of prostate<br>- Erectile dysfunction<br>- Primary malignancy – prostate<br>- Primary malignancy – testicular<br>Women only:<br>- Primary malignancy – cervical<br>- Primary malignancy – ovarian<br>- Primary malignancy – uterine
Lifelong conditions	The following conditions were defined as present from birth irrespective of the date of the earliest record:<br>- Autism and Asperger's syndrome<br>- Intellectual disability<br>- Down's syndrome<br>- Cerebral Palsy<br>- Sickle-cell anaemia<br>- Thalassaemia<br>- Cystic Fibrosis<br>- Juvenile arthritis (sub-condition included within the condition ‘inflammatory arthritis and other inflammatory conditions’)

Supplementary Fig. 3. Flow diagram of sample selection

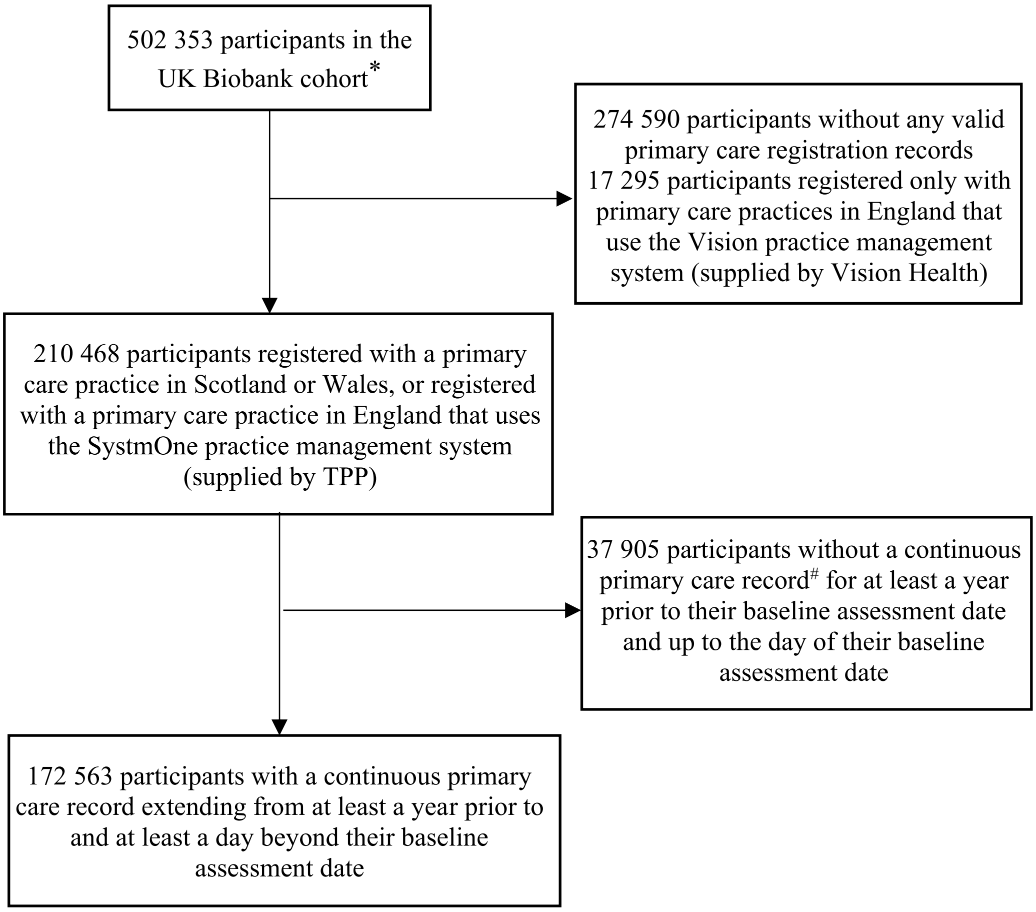

Figure legend: * Excluding participants who withdrew permission for their data to be included in research before 13 October 2023; # We defined a continuous record as one where there was a gap of no more than 90 days between practice registrations.

Supplementary Fig. 4. Proportional Venn diagrams of concordance between data sources for all conditions by body system (using data from all three data sources)

Mental and behavioural disorders

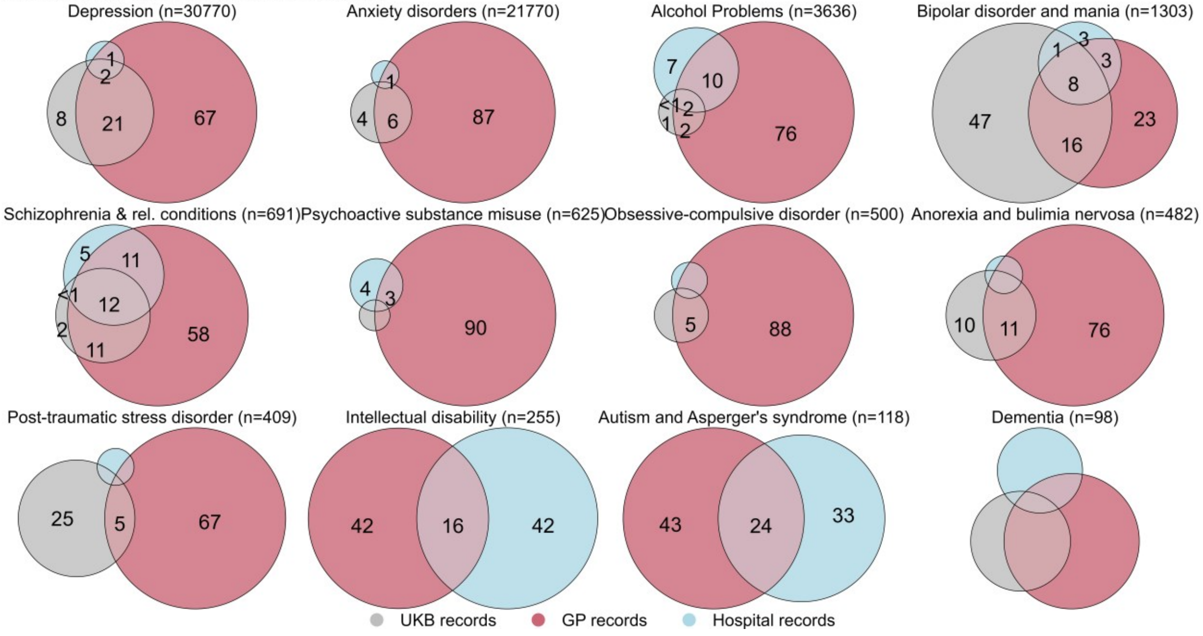

Diseases of the circulatory system

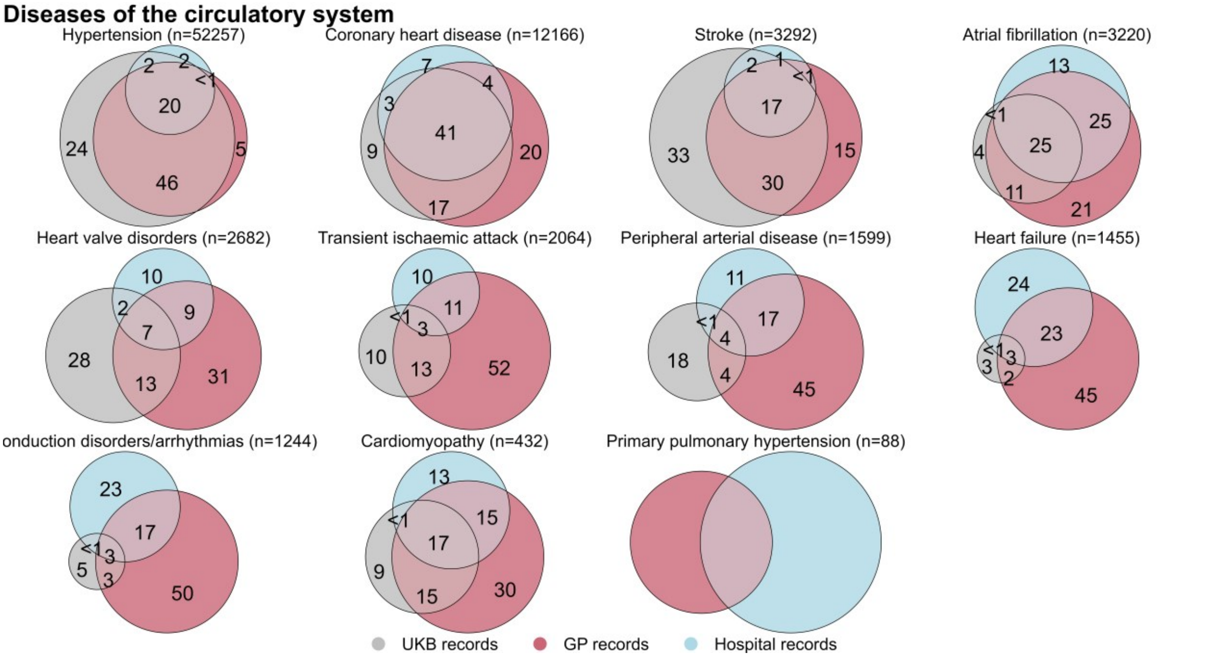

Neoplasms

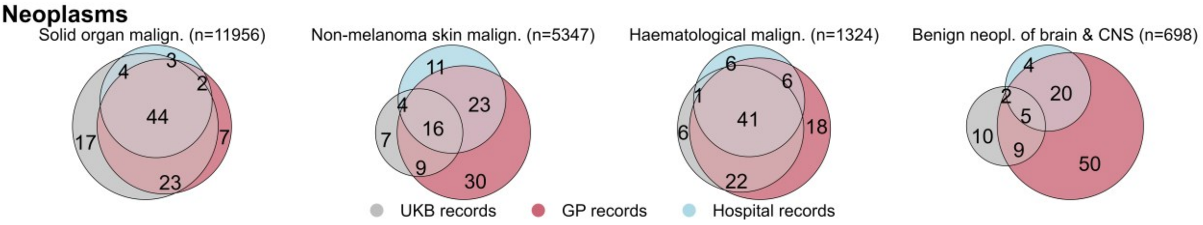

Diseases of the digestive system

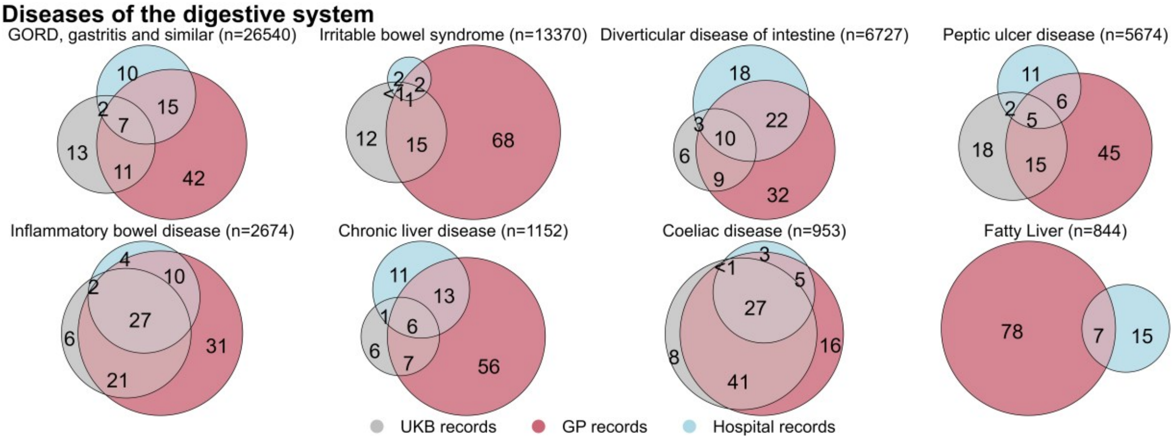

Diseases of the ear and mastoid process

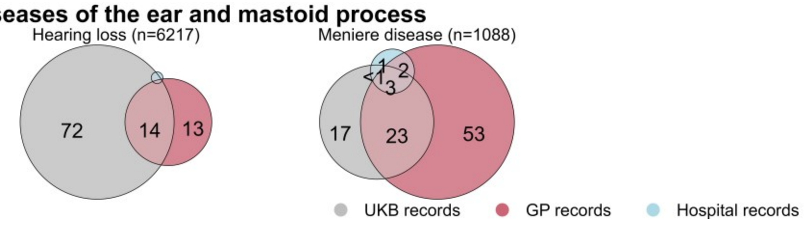

Endocrine, nutritional and metabolic diseases

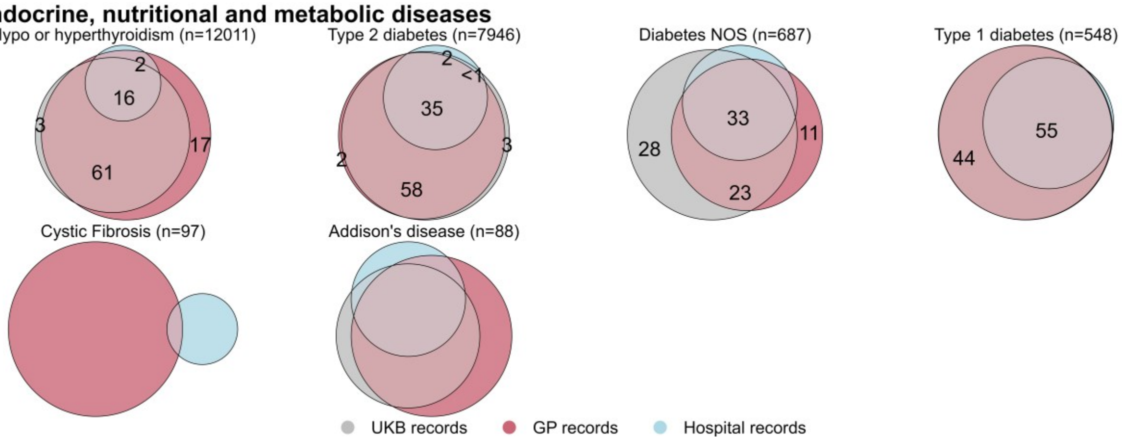

Diseases of the eye and adnexa

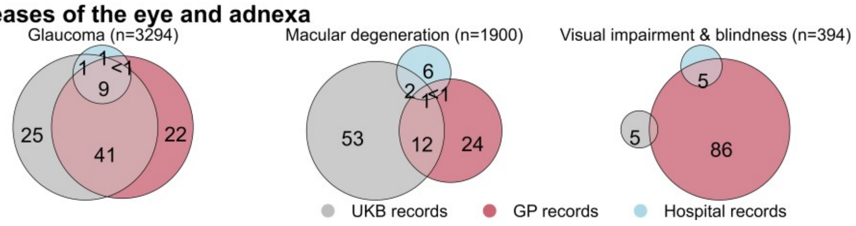

Diseases of the genitourinary system

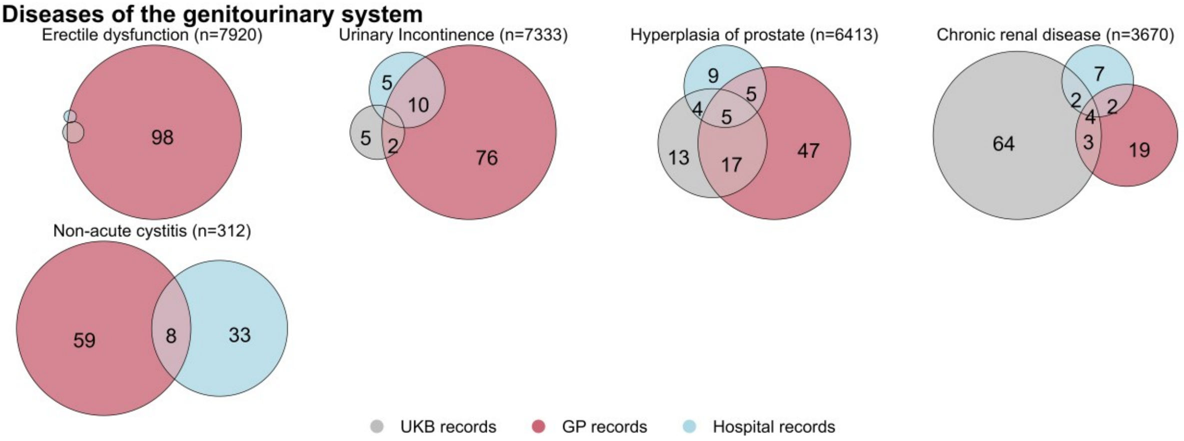

Diseases of the respiratory system

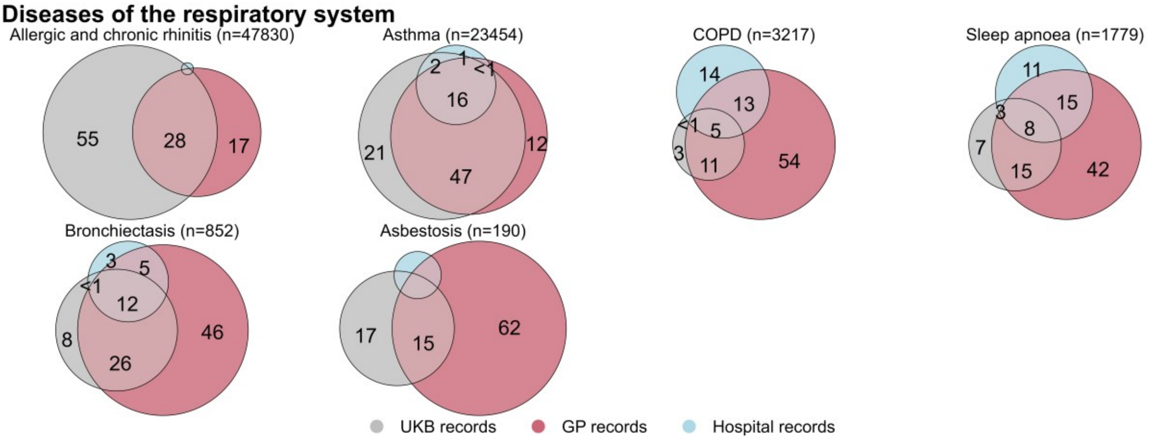

Congenital malformations, deformations and chromosomal abnormalities

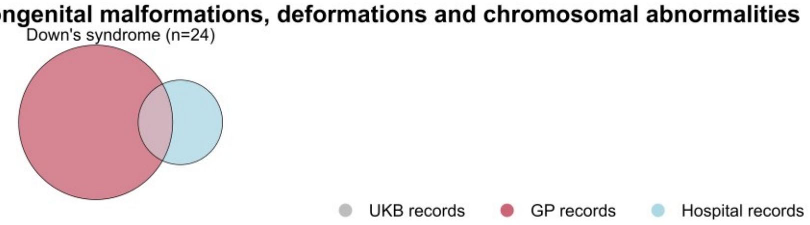

Diseases of the blood and blood-forming organs and certain disorders involving the immune mechanism

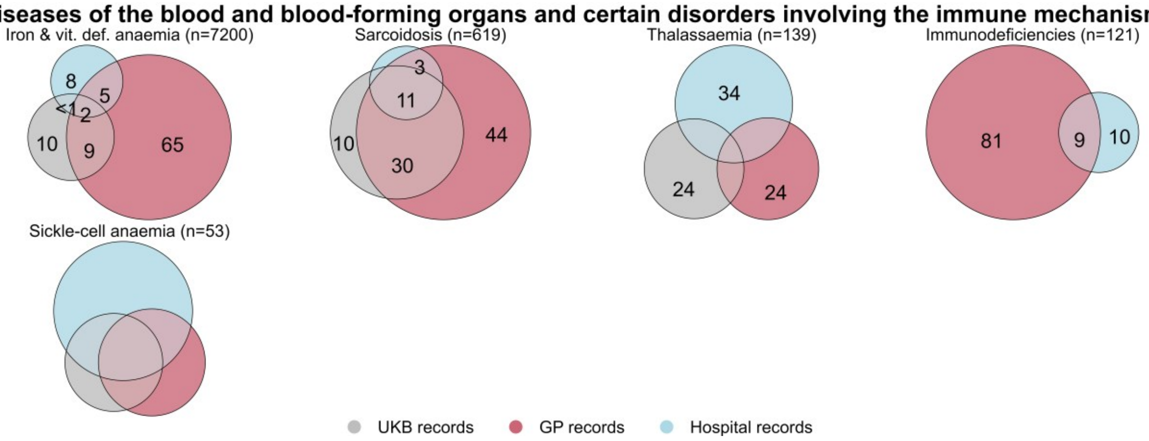

Certain infectious and parasitic diseases

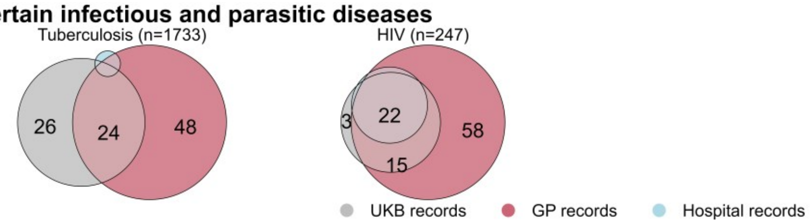

Diseases of the musculoskeletal system and connective tissue

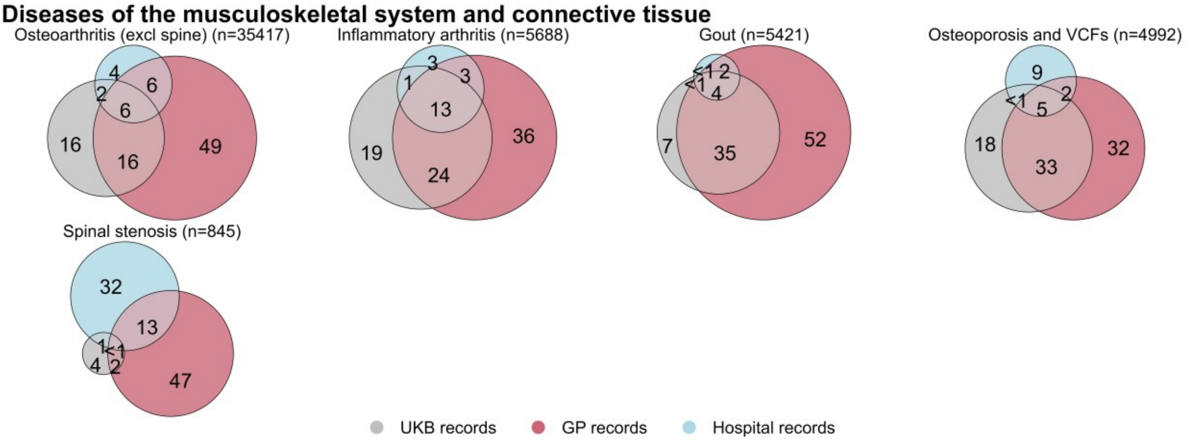

Diseases of the skin and subcutaneous tissue

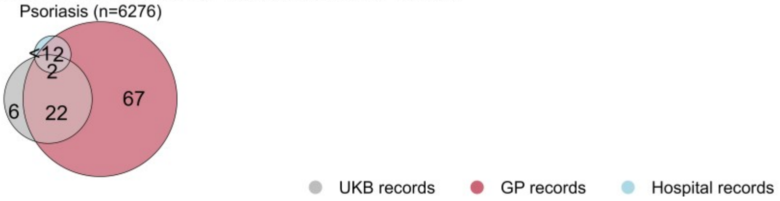

Diseases of the nervous systems

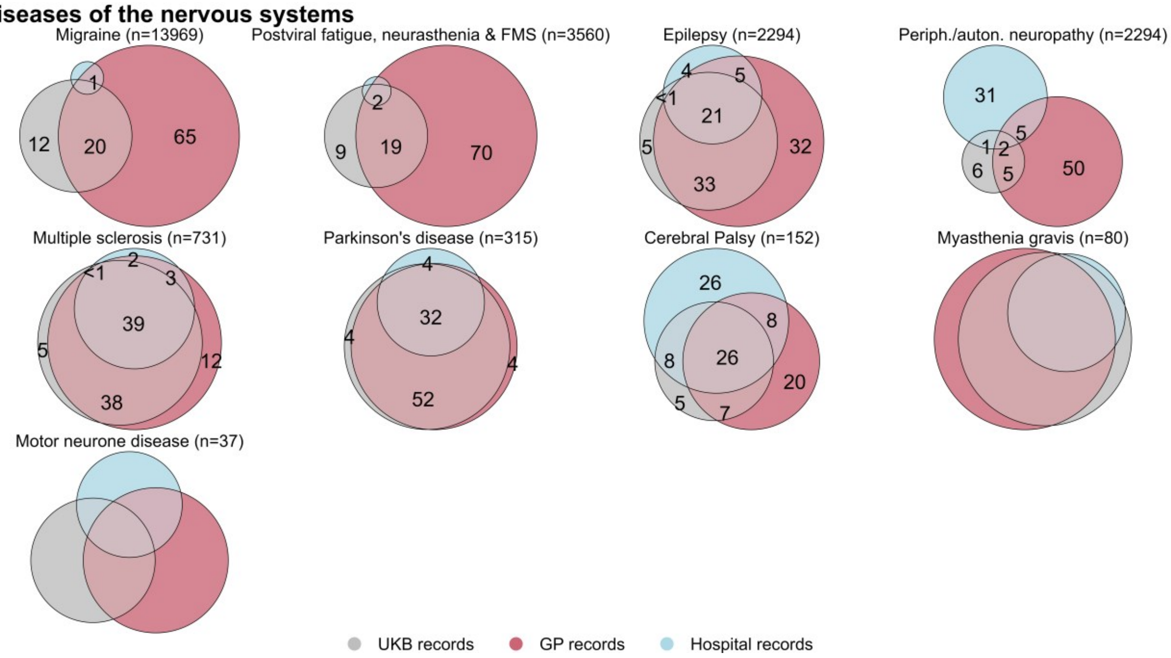

Supplementary Fig. 5. Tornado plot of concordance between primary care and UK Biobank records for all conditions by body system.

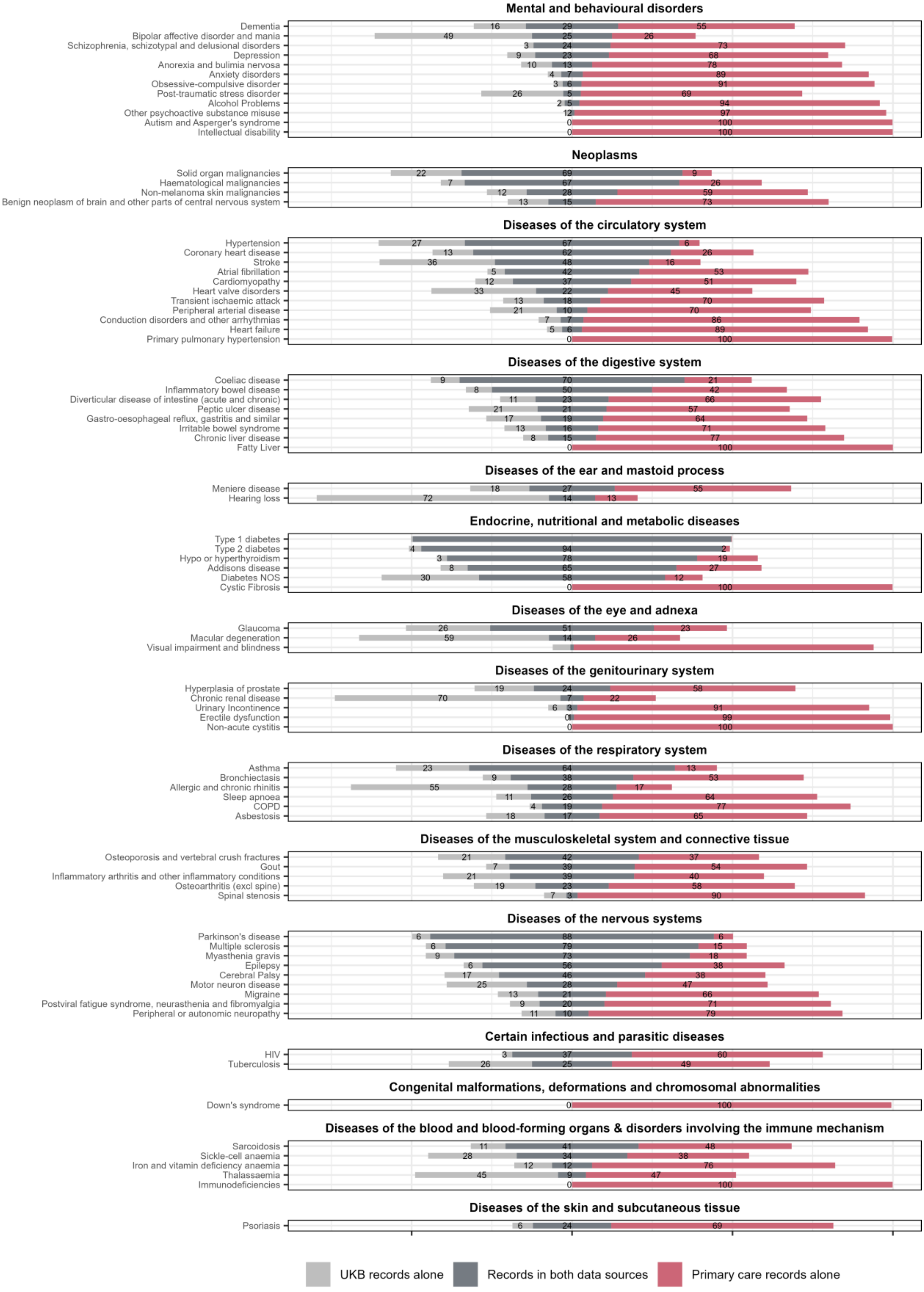

Figure legend: The denominators excluded patients whose conditions were identified in hospital records alone. We have suppressed some cells to avoid disclosing small numbers, in keeping with UK Biobank guidelines

Supplementary Fig. 6. Tornado plot of concordance between primary care and hospital records for all conditions by body system.

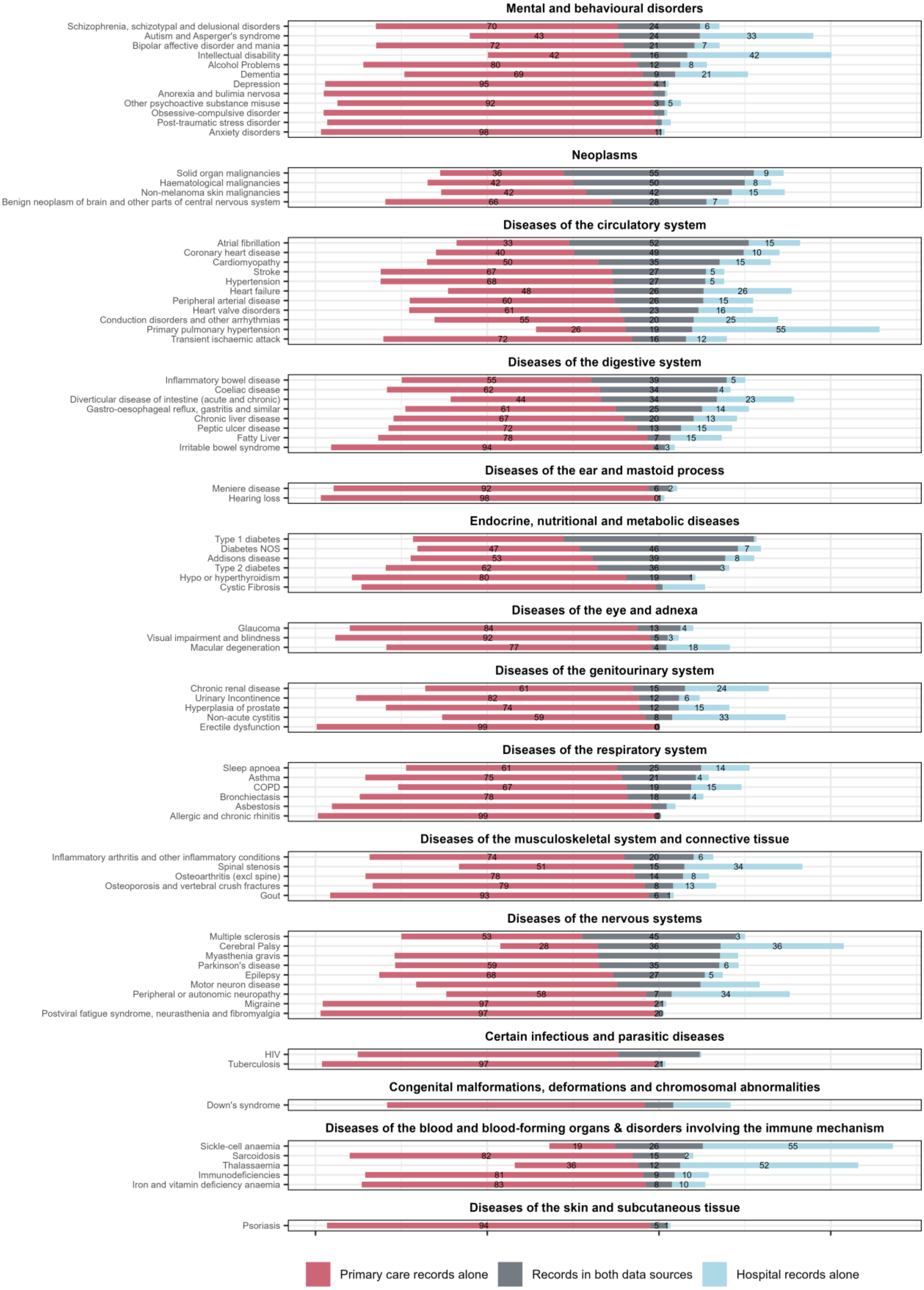

Figure legend: The denominators excluded patients whose conditions were identified in UK Biobank records alone. We have suppressed some cells to avoid disclosing small numbers, in keeping with UK Biobank guidelines

Supplementary Fig. 7. Tornado plot of concordance between UK Biobank records and hospital records for all conditions by body system.

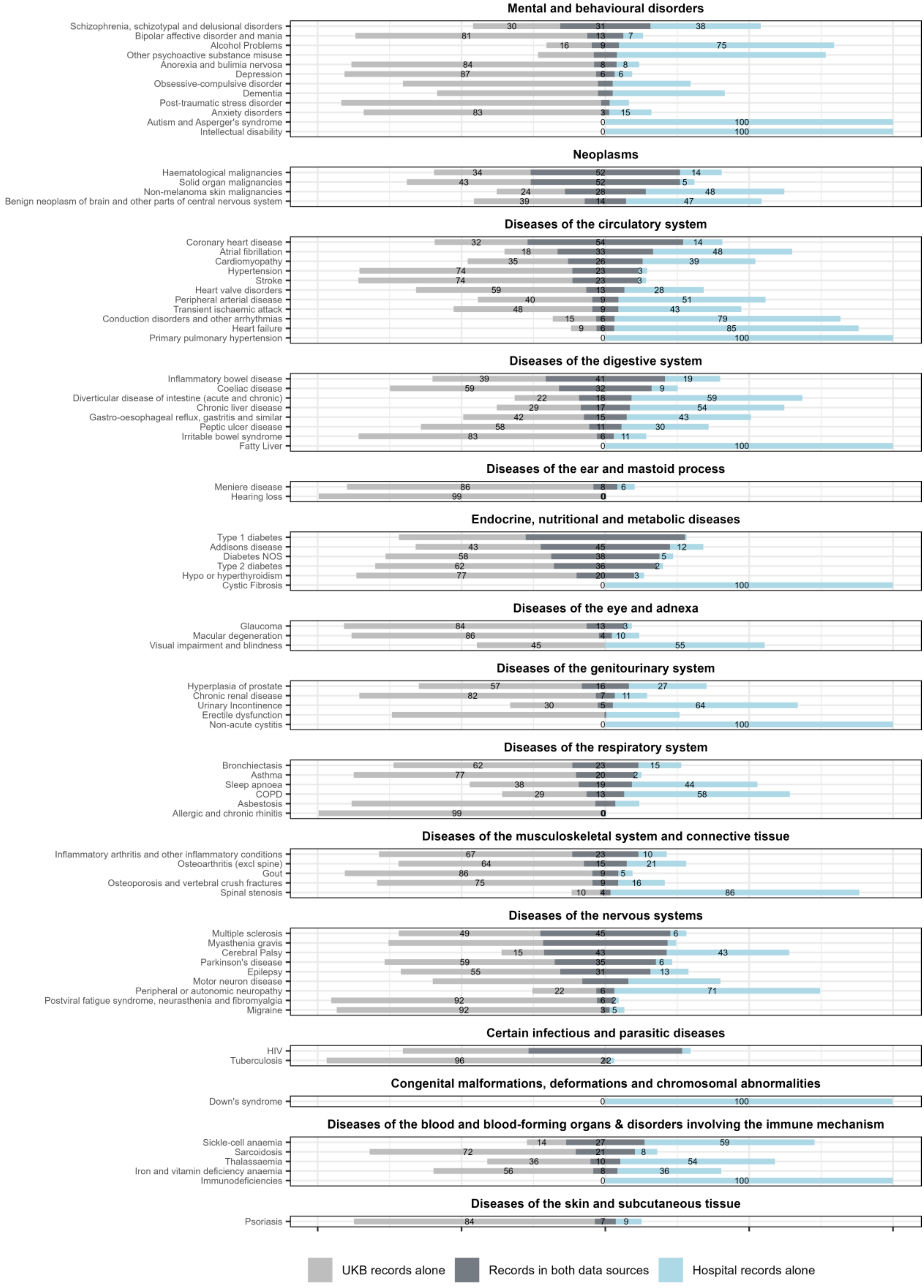

Figure legend: The denominators excluded patients whose conditions were identified in primary care records alone. We have suppressed some cells to avoid disclosing small numbers, in keeping with UK Biobank guidelines

Supplementary Fig. 8. Prevalence of multiple long-term health conditions by age, sex, ethnicity and deprivation for MLTC 3+ and MLTC 3+ from 3+

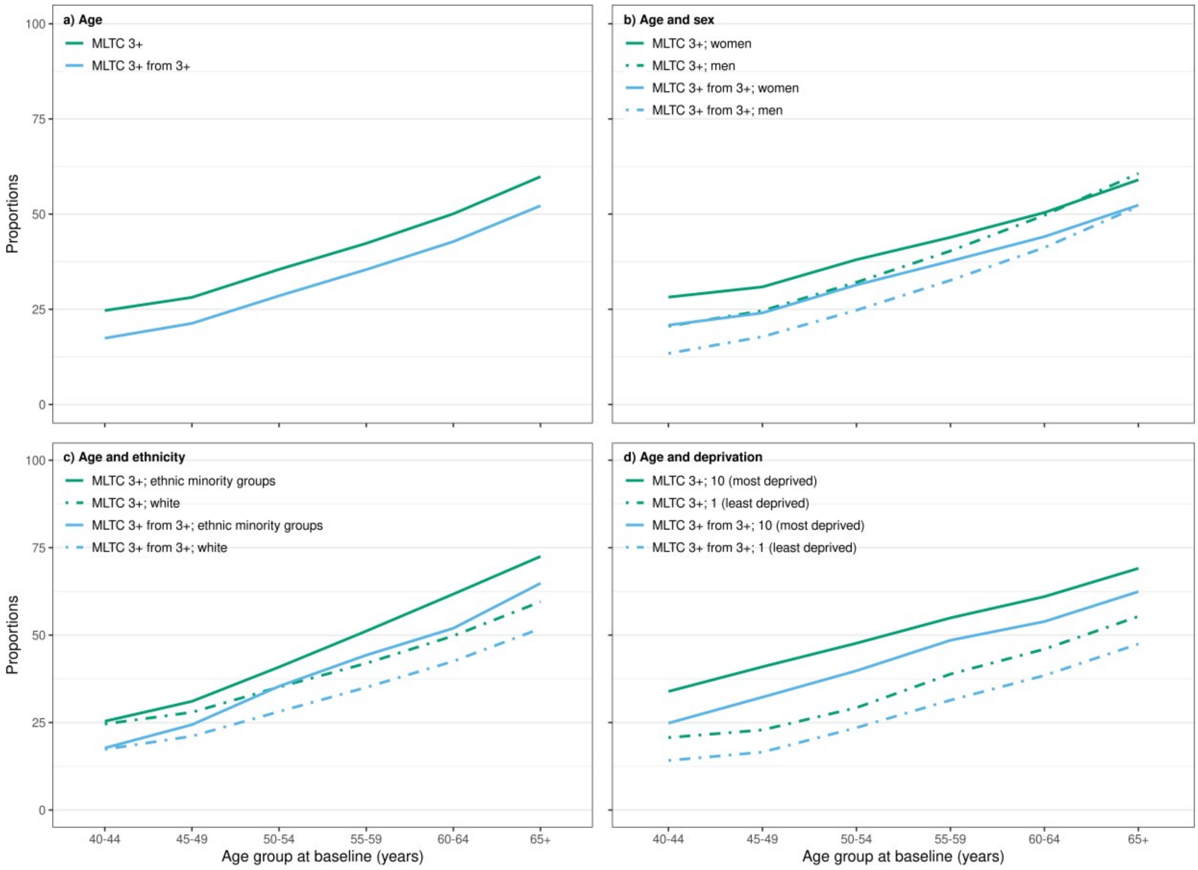

Figure legend: MLTC 3+: ≥ 3 LTCs; MLTC 3+ from 3+: ≥ 3 LTCs from ≥ 3 body systems

Supplementary Table 4. Baseline characteristics of eligible participants for the whole population and for subsets of the population meeting each of the four MLTC definitions (MLTC ascertained using primary care records alone)

	Eligible participants	People with MLTC 2+	People with MLTC 3+	People with MLTC 3+ from 3+	People with mental-physical multimorbidity
**N (% of whole population)**	172563 (100.0)	87750 (50.9)	53977 (31.3)	43547 (25.2)	32557 (18.9)
**Age group, years, (%)**					
40-44	16640 (9.6)	5338 (6.1)	2617 (4.8)	1735 (4.0)	2537 (7.8)
45-49	22351 (13.0)	8020 (9.1)	4096 (7.6)	2994 (6.9)	3581 (11.0)
50-54	26130 (15.1)	11321 (12.9)	6341 (11.7)	4920 (11.3)	4923 (15.1)
55-59	31513 (18.3)	15865 (18.1)	9434 (17.5)	7628 (17.5)	6314 (19.4)
60-64	42372 (24.6)	24627 (28.1)	15770 (29.2)	13080 (30.0)	8611 (26.4)
65+	33557 (19.4)	22579 (25.7)	15719 (29.1)	13190 (30.3)	6591 (20.2)
**Women (%)**	94029 (54.5)	48884 (55.7)	30048 (55.7)	24732 (56.8)	20448 (62.8)
**Ethnicity (%)**					
White	164711 (95.4)	83703 (95.4)	51407 (95.2)	41444 (95.2)	31313 (96.2)
Ethnic minority groups	7109 (4.1)	3631 (4.1)	2306 (4.3)	1886 (4.3)	1099 (3.4)
Missing	743 (0.4)	416 (0.5)	264 (0.5)	217 (0.5)	145 (0.4)
**Townsend index (deciles), (%)***					
1 (least deprived)	17003 (9.9)	8068 (9.2)	4684 (8.7)	3741 (8.6)	2762 (8.5)
2	18230 (10.6)	8798 (10.0)	5243 (9.7)	4227 (9.7)	2982 (9.2)
3	17888 (10.4)	8698 (9.9)	5148 (9.5)	4111 (9.4)	3045 (9.4)
4	16956 (9.8)	8372 (9.5)	5008 (9.3)	4051 (9.3)	2858 (8.8)
5	18121 (10.5)	9098 (10.4)	5551 (10.3)	4445 (10.2)	3298 (10.1)
6	17887 (10.4)	8992 (10.2)	5484 (10.2)	4447 (10.2)	3250 (10.0)
7	17375 (10.1)	8767 (10.0)	5350 (9.9)	4360 (10.0)	3315 (10.2)
8	17219 (10.0)	8974 (10.2)	5632 (10.4)	4504 (10.3)	3437 (10.6)
9	16763 (9.7)	9086 (10.4)	5863 (10.9)	4737 (10.9)	3631 (11.2)
10 (most deprived)	14915 (8.6)	8787 (10.0)	5945 (11.0)	4870 (11.2)	3931 (12.1)
Missing	206 (0.1)	110 (0.1)	69 (0.1)	54 (0.1)	48 (0.1)

Table legend: * Deciles are based on the whole UK Biobank cohort (i.e., also includes people without a continuous primary care record). MLTC: Multiple long-term conditions; MLTC 2+: ≥ 2 long-term conditions (LTCs); MLTC 3+: ≥ 3 LTCs; MLTC 3+ from 3+: ≥ 3 LTCs from ≥ 3 body systems; Mental-physical multimorbidity: ≥ 2 LTCs where ≥ 1 mental health LTC and ≥ 1 physical health LTC

Supplementary Table 5. Baseline characteristics of eligible participants for the whole population and for subsets of the population meeting each of the four MLTC definitions (MLTC ascertained using UKB records alone)

	Eligible participants	People with MLTC 2+	People with MLTC 3+	People with MLTC 3+ from 3+	People with mental-physical multimorbidity
**N (% of whole population)**	172563 (100.0)	67316 (39.0)	30512 (17.7)	23273 (13.5)	9768 (5.7)
**Age group, years, (%)**					
40-44	16640 (9.6)	3983 (5.9)	1325 (4.3)	833 (3.6)	892 (9.1)
45-49	22351 (13.0)	6090 (9.0)	2122 (7.0)	1452 (6.2)	1245 (12.7)
50-54	26130 (15.1)	8553 (12.7)	3448 (11.3)	2561 (11.0)	1620 (16.6)
55-59	31513 (18.3)	12046 (17.9)	5355 (17.6)	4130 (17.7)	2039 (20.9)
60-64	42372 (24.6)	18713 (27.8)	8887 (29.1)	6903 (29.7)	2353 (24.1)
65+	33557 (19.4)	17931 (26.6)	9375 (30.7)	7394 (31.8)	1619 (16.6)
**Women (%)**	94029 (54.5)	37182 (55.2)	17096 (56.0)	13506 (58.0)	6303 (64.5)
**Ethnicity (%)**					
White	164711 (95.4)	64252 (95.4)	29106 (95.4)	22217 (95.5)	9423 (96.5)
Ethnic minority groups	7109 (4.1)	2803 (4.2)	1290 (4.2)	969 (4.2)	307 (3.1)
Missing	743 (0.4)	261 (0.4)	116 (0.4)	87 (0.4)	38 (0.4)
**Townsend index (deciles), (%)***					
1 (least deprived)	17003 (9.9)	6105 (9.1)	2490 (8.2)	1835 (7.9)	730 (7.5)
2	18230 (10.6)	6533 (9.7)	2708 (8.9)	2087 (9.0)	810 (8.3)
3	17888 (10.4)	6727 (10.0)	2854 (9.4)	2158 (9.3)	876 (9.0)
4	16956 (9.8)	6362 (9.5)	2742 (9.0)	2068 (8.9)	811 (8.3)
5	18121 (10.5)	6854 (10.2)	3057 (10.0)	2268 (9.7)	921 (9.4)
6	17887 (10.4)	6828 (10.1)	3061 (10.0)	2335 (10.0)	894 (9.2)
7	17375 (10.1)	6764 (10.0)	3080 (10.1)	2377 (10.2)	987 (10.1)
8	17219 (10.0)	6939 (10.3)	3235 (10.6)	2452 (10.5)	1098 (11.2)
9	16763 (9.7)	7091 (10.5)	3481 (11.4)	2696 (11.6)	1189 (12.2)
10 (most deprived)	14915 (8.6)	7030 (10.4)	3760 (12.3)	2963 (12.7)	1436 (14.7)
Missing	206 (0.1)	83 (0.1)	44 (0.1)	34 (0.1)	16 (0.2)

Table legend: * Deciles are based on the whole UK Biobank cohort (i.e., also includes people without a continuous primary care record). MLTC: Multiple long-term conditions; MLTC 2+: ≥ 2 long-term conditions (LTCs); MLTC 3+: ≥ 3 LTCs; MLTC 3+ from 3+: ≥ 3 LTCs from ≥ 3 body systems; Mental-physical multimorbidity: ≥ 2 LTCs where ≥ 1 mental health LTC and ≥ 1 physical health LTC

Supplementary Table 6. Baseline characteristics of eligible participants for the whole population and for subsets of the population meeting each of the four MLTC definitions (MLTC ascertained using hospital records alone)

	Eligible participants	People with MLTC 2+	People with MLTC 3+	People with MLTC 3+ from 3+	People with mental-physical multimorbidity
**N (% of whole population)**	172563 (100.0)	20661 (12.0)	9416 (5.5)	6018 (3.5)	1855 (1.1)
**Age group at baseline, years, (%)**					
40-44	16640 (9.6)	16640 (9.6)	631 (3.1)	218 (2.3)	145 (2.4)
45-49	22351 (13.0)	22351 (13.0)	1155 (5.6)	449 (4.8)	294 (4.9)
50-54	26130 (15.1)	26130 (15.1)	2061 (10.0)	842 (8.9)	571 (9.5)
55-59	31513 (18.3)	31513 (18.3)	3362 (16.3)	1441 (15.3)	957 (15.9)
60-64	42372 (24.6)	42372 (24.6)	6241 (30.2)	2860 (30.4)	1816 (30.2)
65+	33557 (19.4)	33557 (19.4)	7211 (34.9)	3606 (38.3)	2235 (37.1)
**Women (%)**	94029 (54.5)	94029 (54.5)	9910 (48.0)	4159 (44.2)	2891 (48.0)
**Ethnicity (%)**					
White	164711 (95.4)	164711 (95.4)	19508 (94.4)	8853 (94.0)	5646 (93.8)
Ethnic minority groups	7109 (4.1)	7109 (4.1)	1037 (5.0)	510 (5.4)	339 (5.6)
Missing	743 (0.4)	743 (0.4)	116 (0.6)	53 (0.6)	33 (0.5)
**Townsend index (deciles), (%)***					
1 (least deprived)	17003 (9.9)	17003 (9.9)	1515 (7.3)	577 (6.1)	365 (6.1)
2	18230 (10.6)	18230 (10.6)	1798 (8.7)	741 (7.9)	466 (7.7)
3	17888 (10.4)	17888 (10.4)	1871 (9.1)	767 (8.1)	474 (7.9)
4	16956 (9.8)	16956 (9.8)	1838 (8.9)	791 (8.4)	471 (7.8)
5	18121 (10.5)	18121 (10.5)	1966 (9.5)	865 (9.2)	543 (9.0)
6	17887 (10.4)	17887 (10.4)	2079 (10.1)	961 (10.2)	611 (10.2)
7	17375 (10.1)	17375 (10.1)	2074 (10.0)	957 (10.2)	629 (10.5)
8	17219 (10.0)	17219 (10.0)	2205 (10.7)	1022 (10.9)	654 (10.9)
9	16763 (9.7)	16763 (9.7)	2527 (12.2)	1254 (13.3)	820 (13.6)
10 (most deprived)	14915 (8.6)	14915 (8.6)	2762 (13.4)	1466 (15.6)	978 (16.3)
Missing	206 (0.1)	206 (0.1)	26 (0.1)	15 (0.2)	7 (0.1)

Table legend: * Deciles are based on the whole UK Biobank cohort (i.e., also includes people without a continuous primary care record). MLTC: Multiple long-term conditions; MLTC 2+: ≥ 2 long-term conditions (LTCs); MLTC 3+: ≥ 3 LTCs; MLTC 3+ from 3+: ≥ 3 LTCs from ≥ 3 body systems; Mental-physical multimorbidity: ≥ 2 LTCs where ≥ 1 mental health LTC and ≥ 1 physical health LTC

Supplementary References

1 Lewis, J. D., Bilker, W. B., Weinstein, R. B. & Strom, B. L. The relationship between time since registration and measured incidence rates in the General Practice Research Database. *Pharmacoepidemiol Drug Saf* **14**, 443-451, doi:10.1002/pds.1115 (2005).

2 Kuan, V. *et al.* A chronological map of 308 physical and mental health conditions from 4 million individuals in the English National Health Service. *The Lancet Digital Health* **1**, e63-e77, doi:https://doi.org/10.1016/S2589-7500(19)30012-3 (2019).

3 Ho, I. S. S. *et al.* Measuring multimorbidity in research: Delphi consensus study. *BMJ Medicine* **1**, e000247, doi:10.1136/bmjmed-2022-000247 (2022).

4 Denaxas, S. *et al.* UK phenomics platform for developing and validating electronic health record phenotypes: CALIBER. *Journal of the American Medical Informatics Association*, doi:10.1093/jamia/ocz105 (2019).

5 University of Oxford for the Bennett Institute for Applied Data Science. *OpenSAFELY codelists*, <https://www.opencodelists.org/codelist/opensafely/> (2022).

6 UK Biobank. *Resource 594 - Code lists for algorithmically-defined outcomes*, <https://biobank.ndph.ox.ac.uk/showcase/refer.cgi?id=594> (Accessed: 12/03/2025)

7 Inker Lesley, A. *et al.* New Creatinine- and Cystatin C–Based Equations to Estimate GFR without Race. *New England Journal of Medicine* **385**, 1737-1749, doi:10.1056/NEJMoa2102953 (2021).
