# Supplementary material for: Robustly measuring multimorbidity using disparate linked datasets
Source: Commun Med (Lond). 2025 Jul 8;5:283. doi: 10.1038/s43856-025-00995-4 (PMC12238475; doi:10.1038/s43856-025-00995-4)
Supplement: Supplementary file 3 — Description of Additional Supplementary files [file 43856_2025_995_MOESM3_ESM.pdf]

Description of Additional Supplementary files

File name: Supplementary Data 1

Description: Information used from UK Biobank baseline assessment centre

File name: Supplementary Data 2

Description: List of conditions, sub-conditions, body system and category

File name: Supplementary Data 3

Description: Overview of code lists for each coding system and their sources

File name: Supplementary Data 4

Description: Prevalence of each condition at baseline identified using data from all three data sources, primary care records alone, UKB records alone and hospital records alone

File name: Supplementary Data 5

Description: Numerical results underlying the Venn diagrams of concordance between data sources for all conditions

File name: Supplementary Data 6

Description: Prevalence of multiple long-term health conditions by age, sex, ethnicity and deprivation using four different definitions and records from all three data sources

File name: Supplementary Data 7

Description: Prevalence of multiple long-term health conditions by age using four different definitions and different data sources

File name: Supplementary Data 8

Description: Prevalence of multiple long-term health conditions by age, sex, ethnicity and deprivation using four different definitions and primary care records alone

File name: Supplementary Data 9

Description: Prevalence of multiple long-term health conditions by age, sex, ethnicity and deprivation using four different definitions and UK Biobank records alone

File name: Supplementary Data 10

Description: Prevalence of multiple long-term health conditions by age, sex, ethnicity and deprivation using four different definitions and hospital records alone
