# Supplementary material for: Robustly measuring multimorbidity using disparate linked datasets
Source: Commun Med (Lond). 2025 Jul 8;5:283. doi: 10.1038/s43856-025-00995-4 (PMC12238475; doi:10.1038/s43856-025-00995-4)
Supplement: Supplementary file 4 — Supplementary Data 1 [file 43856_2025_995_MOESM4_ESM.docx]

This supplementary data file outlines which variables were used to identify participants with selected morbidities based on information collected at the baseline assessment centre. The term ‘field ID’ is used to refer to the ID of the corresponding variable in the UK Biobank Data Showcase (<https://biobank.ndph.ox.ac.uk/showcase/>).

## Variables used for a large number of conditions

- Field id 20001 – Cancer code, self-reported – Nurse interview
  - Code for cancer
  - If the participant was uncertain of the type of cancer they had had, then they described it to the interviewer (a trained nurse) who attempted to place it within the coding tree (see https://biobank.ndph.ox.ac.uk/showcase/field.cgi?id=20001).
  - If the cancer could not be located in the coding tree, then the interviewer entered a free-text description of it. These free-text descriptions were subsequently examined by a doctor and, where possible, matched to entries in the coding tree. Free-text descriptions which could not be matched with very high probability have been marked as "unclassifiable".
  - Relevant related field:
    - Field id: 20006: Interpolated Year when cancer first diagnosed
- Field id 20002 – Non-cancer illness code, self-reported – Nurse interview
  - Code for non-cancer illness
  - If the participant was uncertain of the type of illness they had had, then they described it to the interviewer (a trained nurse) who attempted to place it within the coding tree.
  - If the illness could not be located in the coding tree, then the interviewer entered a free-text description of it. These free-text descriptions were subsequently examined by a doctor and, where possible, matched to entries in the coding tree. Free-text descriptions which could not be matched with very high probability have been marked as "unclassifiable".
  - Relevant related field:
    - Field id: 20008: Interpolated Year when non-cancer illness first diagnosed
- Age/ date of diagnosis for each selected morbidity
  - Whenever available, age at diagnosis was set as age at diagnosis reported by participants (in follow-up questions at baseline assessment centre)
  - When age at diagnosis was not assessed or participant did not report age at diagnosis, then the condition was assumed present at baseline and age at diagnosis was set as age when attending the baseline assessment centre
  - Date of diagnosis was approximated based on age at diagnosis, age when attending the baseline assessment centre and date when attending the baseline assessment centre:
    - Calculate difference between age at diagnosis and age when attending baseline assessment centre
    - Date of diagnosis: Subtract age difference in years from date of attending the assessment centre

## Information in UK Biobank baseline assessment for each condition

### Addison’s disease

#### Field id 20002 – Non-cancer illness code, self-reported – Nurse interview

| coding | meaning |
| --- | --- |
| 1234 | adrenocortical insufficiency/addison's disease |

### Alcohol Problems

#### Field id 20002 – Non-cancer illness code, self-reported – Nurse interview

| coding | meaning |
| --- | --- |
| 1408 | alcohol dependency |

### Allergic and chronic rhinitis

#### Field id 6152 - Has a doctor ever told you that you have had any of the following conditions? – Touchscreen

- Possible response options:
  - Blood clot, DVT, bronchitis, emphysema, asthma, rhinitis, eczema, allergy diagnosed by doctor
  - Blood clot in the leg (DVT)
  - Blood clot in the lung
  - Emphysema/chronic bronchitis
  - Asthma
  - Hayfever, allergic rhinitis or eczema
  - None of the above
  - Prefer not to answer
- We identified participants with Allergic and chronic rhinitis if they responded with “Hayfever, allergic rhinitis or eczema”
- Related fields:
  - Field id 3761: Age hay fever, rhinitis or eczema diagnosed

#### Field id 20002 – Non-cancer illness code, self-reported – Nurse interview

| coding | meaning |
| --- | --- |
| 1387 | hayfever/allergic rhinitis |

### Anorexia and bulimia nervosa

#### Field id 20002 – Non-cancer illness code, self-reported – Nurse interview

| coding | meaning |
| --- | --- |
| 1470 | anorexia/bulimia/other eating disorder |

### Anxiety disorders

#### Field id 20002 – Non-cancer illness code, self-reported – Nurse interview

| coding | meaning |
| --- | --- |
| 1287 | anxiety/panic attacks |

### Asbestosis

#### Field id 20002 – Non-cancer illness code, self-reported – Nurse interview

| coding | meaning |
| --- | --- |
| 1120 | asbestosis |

### Asthma

#### Field id 6152 - Has a doctor ever told you that you have had any of the following conditions? – Touchscreen

- Possible response options:
  - Blood clot, DVT, bronchitis, emphysema, asthma, rhinitis, eczema, allergy diagnosed by doctor
  - Blood clot in the leg (DVT)
  - Blood clot in the lung
  - Emphysema/chronic bronchitis
  - Asthma
  - Hayfever, allergic rhinitis or eczema
  - None of the above
  - Prefer not to answer
- Related fields:
  - Field id 3786: Age asthma diagnosed

#### Field id 20002 – Non-cancer illness code, self-reported – Nurse interview

| coding | meaning |
| --- | --- |
| 1111 | asthma |

### Atrial fibrillation

#### Field id 20002 – Non-cancer illness code, self-reported – Nurse interview

| coding | meaning |
| --- | --- |
| 1471 | atrial fibrillation |
| 1483 | atrial flutter |

### Autism and Asperger's syndrome

No information on this morbidity available in UK Biobank baseline assessment

### Benign neoplasm of brain and other parts of central nervous system

#### Field id 20002 – Non-cancer illness code, self-reported – Nurse interview

| coding | meaning |
| --- | --- |
| 1238 | pituitary adenoma/tumour |
| 1659 | meningioma / benign meningeal tumour |
| 1429 | acromegaly |
| 1239 | cushings syndrome |

### Bipolar affective disorder and mania

#### Field id 20002 – Non-cancer illness code, self-reported – Nurse interview

| coding | meaning |
| --- | --- |
| 1291 | mania/bipolar disorder/manic depression |

#### Field id 20126 – Bipolar and major depression status – touchscreen

- Derived variable from Professor Jill Pell from the Institute of Health & Wellbeing, University of Glasgow
- Coding: No bipolar or depression, Bipolar I Disorder, Bipolar II Disorder, Probable recurrent major depression (severe), Probable recurrent major depression (moderate), Single probable major depression episode
- Information on how variable was derived:
  - <https://biobank.ndph.ox.ac.uk/showcase/ukb/docs/TouchscreenQuestionsMainFinal.pdf>
- Bipolar affective disorder and mania was defined as present if participants were allocated to categories ‘Bipolar I disorder’ or ‘Bipolar II disorder’

### Bronchiectasis

#### Field id 20002 – Non-cancer illness code, self-reported – Nurse interview

| coding | meaning |
| --- | --- |
| 1114 | bronchiectasis |

### Cardiomyopathy

#### Field id 20002 – Non-cancer illness code, self-reported – Nurse interview

| coding | meaning |
| --- | --- |
| 1079 | cardiomyopathy |
| 1588 | hypertrophic cardiomyopathy (hcm / hocm) |

### Cerebral Palsy

#### Field id 20002 – Non-cancer illness code, self-reported – Nurse interview

| coding | meaning |
| --- | --- |
| 1433 | cerebral palsy |

### Chronic liver disease

#### Field id 20002 – Non-cancer illness code, self-reported – Nurse interview

| coding | meaning |
| --- | --- |
| 1604 | alcoholic liver disease / alcoholic cirrhosis |
| 1158 | liver failure/cirrhosis |
| 1579 | hepatitis b |
| 1580 | hepatitis c |
| 1141 | oesophageal varicies |
| 1506 | primary biliary cirrhosis |

### Chronic renal disease

#### Field id 20002 – Non-cancer illness code, self-reported – Nurse interview

| coding | meaning |
| --- | --- |
| 1192 | renal/kidney failure |
| 1193 | renal failure requiring dialysis |
| 1194 | renal failure not requiring dialysis |
| 1520 | iga nephropathy |
| 1609 | glomerulnephritis |

#### Field id 30700 – Creatinine

- We used creatinine measurements to define the presence of chronic kidney disease (CKD) at baseline:
  - UK Biobank reports creatinine in μmol/L.
  - We calculated IDMS creatinine from creatinine
    - IDMS creatinine = (IDMS traceable serum creatinine – intercept)/slope
    - Using an intercept of 0.26 and a slope of 1.011
  - We converted IDMS creatinine to mg/dL by multiplying by 0.011312
  - We then calculated estimated glomerular filtration rate (eGFR) using the CKD-EPI 2021 creatinine equation^1^
  - In keeping with the Clinical Practice Guideline for the Evaluation and Management of Chronic Kidney Disease 2024 guideline^2^, we defined CKD based on an eGFR of less than 60 ml per minute per 1.73 m^2^

### Coeliac disease

#### Field id 20002 – Non-cancer illness code, self-reported – Nurse interview

| coding | meaning |
| --- | --- |
| 1456 | malabsorption/coeliac disease |

### Conduction disorders and other arrhythmias

#### Field id 20002 – Non-cancer illness code, self-reported – Nurse interview

| coding | meaning |
| --- | --- |
| 1486 | sick sinus syndrome |
| 1487 | svt / supraventricular tachycardia |
| 1484 | wolff parkinson white / wpw syndrome |

### COPD

#### Field id 20002 – Non-cancer illness code, self-reported – Nurse interview

| coding | meaning |
| --- | --- |
| 1112 | chronic obstructive airways disease/copd |
| 1472 | emphysema |

### Coronary heart disease

#### Field id 6150 - Vascular/heart problems diagnosed by doctor – Touchscreen

- Touchscreen question "Has a doctor ever told you that you have had any of the following conditions?
- Possible responses: Heart attack, stroke, angina, high blood pressure, none of the above, prefer not to answer
- Coronary heart disease was defined as present if participants reported a ‘Heart attack’ or ‘Angina’
- Relevant related fields:
  - Field id 3627: Age angina diagnosed
  - Field id 3894: Age heart attack diagnosed

#### Field id 20002 – Non-cancer illness code, self-reported – Nurse interview

| coding | meaning | individual condition |
| --- | --- | --- |
| 1074 | angina |  |
| 1075 | heart attack/myocardial infarction |  |

### Cystic Fibrosis

No information on this morbidity available in UK Biobank baseline assessment

### Dementia

#### Field id 20002 – Non-cancer illness code, self-reported – Nurse interview

| coding | meaning |
| --- | --- |
| 1263 | dementia/alzheimers/cognitive impairment |

### Depression

#### Field id 20002 – Non-cancer illness code, self-reported – Nurse interview

| coding | meaning |
| --- | --- |
| 1286 | depression |
| 1531 | post-natal depression |

### Diabetes

We defined diabetes type (type 1, type 2 or not otherwise specified) based on the algorithm used by Kuan et al. (<https://doi.org/10.1016/S2589-7500(19)30012-3)>.

- We defined type 1 diabetes if a participant had a record of type 1 diabetes, but no record of type 2 diabetes.
- We defined type 2 diabetes if a participant had a record of type 2 diabetes, but not record of type 1 diabetes.
- We defined diabetes not otherwise specified if a participant had a record of diabetes without the type specified, or if a participant had a mix of type 1 and type 2 diabetes records.

#### Field id 2443 - Diabetes diagnosed by doctor – Touchscreen

- Touchscreen question "Has a doctor ever told you that you have diabetes?"
- Possible responses: Yes, No, Do not know, Prefer not to say
- Participants were coded as having diabetes if they responded with “Yes” and did not respond with “Yes” to field id 4041: Gestational diabetes only
- Other relevant related fields:
  - Field id 2976: Age diabetes diagnosed

#### Field id 20002 – Non-cancer illness code, self-reported – Nurse interview

| coding | meaning |
| --- | --- |
| 1220 | diabetes |
| 1222 | type 1 diabetes |
| 1223 | type 2 diabetes |

#### Field id 6148: Eye problems/disorders

- Touchscreen question "Has a doctor told you that you have any of the following problems with your eyes?
- Possible response: Diabetes related eye disease
- Decided to add this information because, out of 3706 participants that indicated that they had diabetes related eye disease at baseline, 46 participants were not diagnosed with diabetes based on any other relevant variable at baseline.
- Relevant related fields:
  - Field id 5901: Age when diabetes-related eye disease diagnosed

### Diverticular disease of intestine (acute and chronic)

#### Field id 20002 – Non-cancer illness code, self-reported – Nurse interview

| coding | meaning |
| --- | --- |
| 1458 | diverticular disease/diverticulitis |

### Down's syndrome

No information on this morbidity available in UK Biobank baseline assessment

### Epilepsy

#### Field id 20002 – Non-cancer illness code, self-reported – Nurse interview

| coding | meaning |
| --- | --- |
| 1264 | epilepsy |

### Erectile dysfunction

We checked whether participants with erectile dysfunction were male. All participants with erectile dysfunction were male.

#### Field id 20002 – Non-cancer illness code, self-reported – Nurse interview

| coding | meaning |
| --- | --- |
| 1518 | erectile dysfunction / impotence |

### Fatty Liver

No information on this morbidity available in UK Biobank baseline assessment

### Gastro-oesophageal reflux, gastritis and similar

#### Field id 20002 – Non-cancer illness code, self-reported – Nurse interview

| coding | meaning |
| --- | --- |
| 1138 | gastro-oesophageal reflux (gord) / gastric reflux |
| 1139 | oesophagitis/barretts oesophagus |

### Glaucoma

#### Field id 5326 - Ever had surgery for glaucoma or high eye pressure

- If the participant stated they had eye surgery (Field id 5181), they were asked if they had ever had surgery for glaucoma or high eye pressure. If yes, they were excluded from all eye tests.
- Possible response: No, Right eye only, Left eye only, Both eyes, Do not know
- Participants were coded as having glaucoma if they responded with:
  - Right eye only or
  - Left eye only or
  - Both eyes

#### Field id 5327 - Ever had laser treatment for glaucoma or high eye pressure

- If the participant stated they had eye surgery (Field id 5181), they were asked if they had ever had laser treatment for glaucoma or high eye pressure. If yes, they were excluded from all eye tests.
- Possible response: No, Right eye only, Left eye only, Both eyes, Do not know
- Participants were coded as having glaucoma if they responded with:
  - Right eye only or
  - Left eye only or
  - Both eyes

#### Field id 6148 - Eye problems/disorders – Touchscreen

- Touchscreen question "Has a doctor told you that you have any of the following problems with your eyes? (You can select more than one answer)"
- Possible responses: Diabetes related eye disease, Glaucoma, Injury or trauma resulting in loss of vision, cataract, macular degeneration, other serious eye conditions, none of the above, prefer not to answer, do not know
- Participants were coded as having glaucoma if they responded with “Glaucoma”
- See variable 6119 to determine which eye(s) affected

#### Field id 20002 – Non-cancer illness code, self-reported – Nurse interview

| coding | meaning |
| --- | --- |
| 1277 | glaucoma |

### Gout

#### Field id 20002 – Non-cancer illness code, self-reported – Nurse interview

| coding | meaning |
| --- | --- |
| 1466 | gout |

### Haematological malignancies

#### Field id 20001 – Cancer code, self-reported – Nurse interview

| coding | meaning |
| --- | --- |
| 1047 | lymphoma |
| 1048 | leukaemia |
| 1050 | multiple myeloma |
| 1051 | myelofibrosis or myelodysplasia |
| 1052 | hodgkins lymphoma / hodgkins disease |
| 1053 | non-hodgkins lymphoma |
| 1055 | chronic lymphocytic |
| 1056 | chronic myeloid |
| 1058 | other haematological malignancy |
| 1074 | acute myeloid leukaemia |

#### Field id 20002 – Non-cancer illness code, self-reported – Nurse interview

| coding | meaning |
| --- | --- |
| 1438 | polycythaemia vera |
| 1450 | monoclonal gammopathy/not myeloma |
| 1449 | myeloproliferative disorder |
| 1658 | myelofibrosis |

### Hearing loss

#### Field id 2247 - Hearing difficulty/problems – Touchscreen

- Touchscreen question "Do you have any difficulty with your hearing?"
- Possible responses: Yes, No, I am completely deaf, Do not know, Prefer not to say
- Participants were coded as having hearing loss if they responded with “I am completely deaf”

#### Field id 3393 - Hearing aid user - Touchscreen

- Initially this information was collected from all participants answering Yes to either Field 2247 (Field 10793 in the pilot) or Field 2257. When the speech-in-noise hearing test was introduced (2009), program logic was altered so that the information was collected from all participants except those who indicated they were completely deaf, as defined by their answers to Field 2247
- Touchscreen question: "Do you use a hearing aid most of the time?"
- Participants were coded as having hearing loss if they responded with “Yes”

#### Field id 4792 - Cochlear implant

- Touchscreen question "Do you have a cochlear implant?"
- Collected from all participants except those who indicated they were completely deaf
- Participants were coded as having hearing loss if they responded with “Yes”

### Heart failure

#### Field id 20002 – Non-cancer illness code, self-reported – Nurse interview

| coding | meaning |
| --- | --- |
| 1076 | heart failure/pulmonary odema |

### Heart valve disorders

#### Field id 20002 – Non-cancer illness code, self-reported – Nurse interview

| coding | meaning |
| --- | --- |
| 1078 | heart valve problem/heart murmur |
| 1488 | mitral valve prolapse |
| 1489 | mitral stenosis |
| 1490 | aortic stenosis |
| 1584 | mitral valve disease |
| 1585 | mitral regurgitation / incompetence |
| 1586 | aortic valve disease |
| 1587 | aortic regurgitation / incompetence |

### HIV

#### Field id 20002 – Non-cancer illness code, self-reported – Nurse interview

| coding | meaning |
| --- | --- |
| 1439 | hiv/aids |

### Hyperplasia of prostate

We checked whether participants with hyperplasia of prostate were male. All participants with hyperplasia of prostate were male.

#### Field id 20002 – Non-cancer illness code, self-reported – Nurse interview

| coding | meaning |
| --- | --- |
| 1396 | enlarged prostate |
| 1516 | bph / benign prostatic hypertrophy |

### Hypertension

#### Field id 6150 - Vascular/heart problems diagnosed by doctor – Touchscreen

- Touchscreen question "Has a doctor ever told you that you have had any of the following conditions?
  - Possible responses: Heart attack, stroke, angina, high blood pressure, none of the above, prefer not to answer
- Participants were identified with hypertension if they responded with “High blood pressure”
- Related field:
  - Field id 2966: Age high blood pressure diagnosed

#### Field id 20002 – Non-cancer illness code, self-reported – Nurse interview

| coding | meaning |
| --- | --- |
| 1065 | hypertension |
| 1072 | essential hypertension |

### Hypo or hyperthyroidism

#### Field id 20002 – Non-cancer illness code, self-reported – Nurse interview

| coding | meaning |
| --- | --- |
| 1228 | thyroid radioablation therapy |
| 1225 | hyperthyroidism/thyrotoxicosis |
| 1226 | hypothyroidism/myxoedema |
| 1428 | thyroiditis |
| 1522 | grave's disease |

### Immunodeficiencies

No information on this morbidity available in UK Biobank baseline assessment

### Inflammatory arthritis and other inflammatory conditions

#### Field id 20002 – Non-cancer illness code, self-reported – Nurse interview

| coding | meaning |
| --- | --- |
| 1313 | ankylosing spondylitis |
| 1377 | polymyalgia rheumatica |
| 1381 | systemic lupus erythematosis/sle |
| 1384 | scleroderma/systemic sclerosis |
| 1464 | rheumatoid arthritis |
| 1477 | psoriatic arthropathy |

### Inflammatory bowel disease

#### Field id 20002 – Non-cancer illness code, self-reported – Nurse interview

| coding | meaning |
| --- | --- |
| 1461 | inflammatory bowel disease |
| 1462 | crohns disease |
| 1463 | ulcerative colitis |

### Intellectual disability

No information on this morbidity available in UK Biobank baseline assessment

### Iron and vitamin deficiency anaemia

#### Field id 20002 – Non-cancer illness code, self-reported – Nurse interview

| coding | meaning |
| --- | --- |
| 1330 | iron deficiency anaemia |
| 1331 | pernicious anaemia |

### Irritable bowel syndrome

#### Field id 20002 – Non-cancer illness code, self-reported – Nurse interview

| coding | meaning |
| --- | --- |
| 1154 | irritable bowel syndrome |

### Macular degeneration

#### Field id 6148 - Eye problems/disorders – Touchscreen

- Touchscreen question "Has a doctor told you that you have any of the following problems with your eyes? (You can select more than one answer)"
- Possible responses: Diabetes related eye disease, Glaucoma, Injury or trauma resulting in loss of vision, cataract, macular degeneration, other serious eye conditions, none of the above, prefer not to answer, do not know
- Participants were identified with Macular degeneration if they selected the response option ‘Macular degeneration’

#### Field id 20002 – Non-cancer illness code, self-reported – Nurse interview

| coding | meaning |
| --- | --- |
| 1528 | macular degeneration |

### Meniere disease

#### Field id 20002 – Non-cancer illness code, self-reported – Nurse interview

| coding | meaning |
| --- | --- |
| 1421 | meniere's disease |

### Migraine

#### Field id 20002 – Non-cancer illness code, self-reported – Nurse interview

| coding | meaning |
| --- | --- |
| 1265 | migraine |

### Motor neurone disease

#### Field id 20002 – Non-cancer illness code, self-reported – Nurse interview

| coding | meaning |
| --- | --- |
| 1259 | motor neurone disease |

### Multiple sclerosis

#### Field id 20002 – Non-cancer illness code, self-reported – Nurse interview

| coding | meaning |
| --- | --- |
| 1261 | multiple sclerosis |

### Myasthenia gravis

#### Field id 20002 – Non-cancer illness code, self-reported – Nurse interview

Info on UKB website: Note that myasthenia gravis appears twice (under codes 1260 and 1437). Please ensure you use both codes to capture all relevant diagnoses.

| coding | meaning |
| --- | --- |
| 1260 | myasthenia gravis |
| 1437 | myasthenia gravis |

### Non-acute cystitis

No information on this morbidity available in UK Biobank baseline assessment

### Non-melanoma skin malignancies

#### Field id 20001 – Cancer code, self-reported – Nurse interview

| coding | meaning |
| --- | --- |
| 1060 | non-melanoma skin cancer |
| 1061 | basal cell carcinoma |
| 1073 | rodent ulcer |

#### Field id 20002 – Non-cancer illness code, self-reported – Nurse interview

| coding | meaning |
| --- | --- |
| 1680 | bowen's disease |

### Obsessive-compulsive disorder

#### Field id 20002 – Non-cancer illness code, self-reported – Nurse interview

| coding | meaning |
| --- | --- |
| 1615 | obsessive compulsive disorder (ocd) |

### Osteoarthritis (excl spine)

#### Field id 20002 – Non-cancer illness code, self-reported – Nurse interview

| coding | meaning |
| --- | --- |
| 1465 | osteoarthritis |

### Osteoporosis and vertebral crush fractures

#### Field id 20002 – Non-cancer illness code, self-reported – Nurse interview

| coding | meaning |
| --- | --- |
| 1309 | osteoporosis |
| 1646 | fracture vertebra / crush fracture / vertebral collapse |

### Other psychoactive substance misuse

#### Field id 20002 – Non-cancer illness code, self-reported – Nurse interview

| coding | meaning |
| --- | --- |
| 1409 | opioid dependency |
| 1410 | other substance abuse/dependency |

### Parkinson's disease

#### Field id 20002 – Non-cancer illness code, self-reported – Nurse interview

| coding | meaning |
| --- | --- |
| 1262 | parkinsons disease |

### Peptic ulcer disease

#### Field id 20002 – Non-cancer illness code, self-reported – Nurse interview

| coding | meaning |
| --- | --- |
| 1142 | gastric/stomach ulcers |
| 1400 | peptic ulcer |
| 1457 | duodenal ulcer |

### Peripheral arterial disease

#### Field id 20002 – Non-cancer illness code, self-reported – Nurse interview

| coding | meaning |
| --- | --- |
| 1067 | peripheral vascular disease |
| 1087 | leg claudication/ intermittent claudication |

### Peripheral or autonomic neuropathy

#### Field id 20002 – Non-cancer illness code, self-reported – Nurse interview

| coding | meaning |
| --- | --- |
| 1468 | diabetic neuropathy/ulcers |
| 1255 | peripheral neuropathy |
| 1256 | acute infective polyneuritis/guillain-barre syndrome |

### Post-traumatic stress disorder

#### Field id 20002 – Non-cancer illness code, self-reported – Nurse interview

| coding | meaning |
| --- | --- |
| 1469 | post-traumatic stress disorder |

### Postviral fatigue syndrome, neurasthenia and fibromyalgia

#### Field id 20002 – Non-cancer illness code, self-reported – Nurse interview

| coding | meaning |
| --- | --- |
| 1482 | chronic fatigue syndrome |
| 1542 | fibromyalgia |

### Primary pulmonary hypertension

No information on this morbidity available in UK Biobank baseline assessment

### Psoriasis

#### Field id 20002 – Non-cancer illness code, self-reported – Nurse interview

| coding | meaning |
| --- | --- |
| 1453 | psoriasis |

### Sarcoidosis

#### Field id 20002 – Non-cancer illness code, self-reported – Nurse interview

| coding | meaning |
| --- | --- |
| 1371 | sarcoidosis |

### Schizophrenia, schizotypal and delusional disorders

#### Field id 20002 – Non-cancer illness code, self-reported – Nurse interview

| coding | meaning |
| --- | --- |
| 1289 | schizophrenia |

### Sickle-cell anaemia

#### Field id 20002 – Non-cancer illness code, self-reported – Nurse interview

| coding | meaning |
| --- | --- |
| 1339 | sickle cell disease |

### Sleep apnoea

#### Variable 20002 – Non-cancer illness code, self-reported – Nurse interview

| coding | meaning |
| --- | --- |
| 1123 | sleep apnoea |

### Solid organ malignancies

#### Field id 20001 – Cancer code, self-reported – Nurse interview

We checked whether participants with male genital tract cancer, penis cancer, prostate cancer or testicular cancer were male. Likewise, we checked whether participants with cervical cancer, fallopian tube cancer, female genital tract cancer, ovarian cancer, uterine/endometrial cancer, vaginal cancer or vulval cancer were female. All records of these cancers were consistent with participant sex.

| coding | meaning |
| --- | --- |
| 1001 | lung cancer |
| 1002 | breast cancer |
| 1004 | cancer of lip/mouth/pharynx/oral cavity |
| 1005 | salivary gland cancer |
| 1006 | larynx/throat cancer |
| 1007 | nasal cavity cancer |
| 1008 | ear cancer |
| 1009 | sinus cancer |
| 1010 | lip cancer |
| 1011 | tongue cancer |
| 1012 | gum cancer |
| 1015 | parotid gland cancer |
| 1016 | other salivary gland cancer |
| 1017 | oesophageal cancer |
| 1018 | stomach cancer |
| 1019 | small intestine/small bowel cancer |
| 1020 | large bowel cancer/colorectal cancer |
| 1021 | anal cancer |
| 1022 | colon cancer/sigmoid cancer |
| 1023 | rectal cancer |
| 1024 | liver/hepatocellular cancer |
| 1025 | gallbladder/bile duct cancer |
| 1026 | pancreas cancer |
| 1027 | small cell lung cancer |
| 1028 | non-small cell lung cancer |
| 1029 | peripheral nerve/autonomic nerve cancer |
| 1030 | eye and/or adnexal cancer |
| 1031 | meningeal cancer / malignant meningioma |
| 1032 | brain cancer / primary malignant brain tumour |
| 1033 | spinal cord or cranial nerve cancer |
| 1034 | kidney/renal cell cancer |
| 1035 | bladder cancer |
| 1036 | other cancer of urinary tract |
| 1037 | female genital tract cancer |
| 1038 | male genital tract cancer |
| 1039 | ovarian cancer |
| 1040 | uterine/endometrial cancer |
| 1041 | cervical cancer |
| 1042 | vaginal cancer |
| 1043 | vulval cancer |
| 1044 | prostate cancer |
| 1045 | testicular cancer |
| 1046 | penis cancer |
| 1059 | malignant melanoma |
| 1062 | squamous cell carcinoma |
| 1063 | primary bone cancer |
| 1064 | mesothelioma |
| 1065 | thyroid cancer |
| 1066 | parathyroid cancer |
| 1067 | adrenal cancer |
| 1068 | sarcoma/fibrosarcoma |
| 1070 | malignant lymph node, unspecified |
| 1071 | metastatic cancer (unknown primary) |
| 1075 | retinoblastoma |
| 1077 | mouth cancer |
| 1078 | tonsil cancer |
| 1079 | oropharynx / oropharyngeal cancer |
| 1080 | trachea cancer |
| 1081 | thymus cancer / malignant thymoma |
| 1082 | heart / mediastinum cancer |
| 1084 | respiratory / intrathoracic cancer |
| 1085 | bone metastases / bony secondaries |
| 1086 | appendix cancer |
| 1087 | fallopian tube cancer |
| 1088 | malignant insulinoma |

### Spinal stenosis

#### Field id 20002 – Non-cancer illness code, self-reported – Nurse interview

| coding | meaning |
| --- | --- |
| 1536 | spinal stenosis |

### Stroke

#### Field id 6150 - Vascular/heart problems diagnosed by doctor – Touchscreen

- Touchscreen question "Has a doctor ever told you that you have had any of the following conditions?
- Possible responses: Heart attack, stroke, angina, high blood pressure, none of the above, prefer not to answer
- Participants were identified with stroke if they selected the response option “Stroke”
- Relevant related fields:
  - Field id 4056: Age stroke diagnosed

#### Field id 20002 – Non-cancer illness code, self-reported – Nurse interview

| coding | meaning |
| --- | --- |
| 1081 | stroke |
| 1583 | ischaemic stroke |

### Thalassaemia

#### Field id 20002 – Non-cancer illness code, self-reported – Nurse interview

| coding | meaning |
| --- | --- |
| 1340 | thalassaemia |

### Transient ischaemic attack

#### Field id 20002 – Non-cancer illness code, self-reported – Nurse interview

| coding | meaning |
| --- | --- |
| 1082 | transient ischaemic attack (tia) |

### Tuberculosis

#### Field id 20002 – Non-cancer illness code, self-reported – Nurse interview

| coding | meaning |
| --- | --- |
| 1440 | tuberculosis (tb) |

### Urinary Incontinence

#### Field id 20002 – Non-cancer illness code, self-reported – Nurse interview

| coding | meaning |
| --- | --- |
| 1202 | urinary frequency / incontinence |

### Visual impairment and blindness

#### Field id 6148 - Eye problems/disorders – Touchscreen

- Touchscreen question "Has a doctor told you that you have any of the following problems with your eyes? (You can select more than one answer)"
- Possible responses: Diabetes related eye disease, Glaucoma, Injury or trauma resulting in loss of vision, cataract, macular degeneration, other serious eye conditions, none of the above, prefer not to answer, do not know
- Participants were identified with visual impairment if:
  - They selected “Injury or trauma resulting in loss of vision” AND
  - They indicated that it resulted in loss of vision of both eyes (Field id: 5419)
    - Touchscreen question "Which eye(s) are affected by injury or trauma resulting in loss of vision?"
      - Possible responses: Left eye, Right eye, Both eyes
- Other relevant related fields:
  - Field id 5430: Age when loss of vision due to injury diagnosed

# References

1 Inker Lesley, A. *et al.* New Creatinine- and Cystatin C–Based Equations to Estimate GFR without Race. *New England Journal of Medicine* **385**, 1737-1749, doi:10.1056/NEJMoa2102953 (2021).

2 Kidney Disease: Improving Global Outcomes (KDIGO) CKD Work Group. KDIGO 2024 Clinical Practice Guideline for the Evaluation and Management of Chronic Kidney Disease. *Kidney Int* **105**, S117-s314, doi:10.1016/j.kint.2023.10.018 (2024).
