# Supplementary material for: Robustly measuring multimorbidity using disparate linked datasets
Source: Commun Med (Lond). 2025 Jul 8;5:283. doi: 10.1038/s43856-025-00995-4 (PMC12238475; doi:10.1038/s43856-025-00995-4)
Supplement: Supplementary file 14 — Reporting summary [file 43856_2025_995_MOESM14_ESM.pdf]

nature portfolio

Corresponding author(s): Prigge, Regina

Last updated by author(s): Mar 4, 2025

Reporting Summary

Nature Portfolio wishes to improve the reproducibility of the work that we publish. This form provides structure for consistency and transparency in reporting. For further information on Nature Portfolio policies, see our Editorial Policies and the Editorial Policy Checklist.

Statistics

For all statistical analyses, confirm that the following items are present in the figure legend, table legend, main text, or Methods section.

n/a	Confirmed	
☐	☒	The exact sample size (*n*) for each experimental group/condition, given as a discrete number and unit of measurement
☐	☒	A statement on whether measurements were taken from distinct samples or whether the same sample was measured repeatedly
☒	☐	The statistical test(s) used AND whether they are one- or two-sided<br>*Only common tests should be described solely by name; describe more complex techniques in the Methods section.*
☐	☒	A description of all covariates tested
☒	☐	A description of any assumptions or corrections, such as tests of normality and adjustment for multiple comparisons
☐	☒	A full description of the statistical parameters including central tendency (e.g. means) or other basic estimates (e.g. regression coefficient) AND variation (e.g. standard deviation) or associated estimates of uncertainty (e.g. confidence intervals)
☒	☐	For null hypothesis testing, the test statistic (e.g. *F*, *t*, *r*) with confidence intervals, effect sizes, degrees of freedom and *P* value noted<br>*Give P values as exact values whenever suitable.*
☒	☐	For Bayesian analysis, information on the choice of priors and Markov chain Monte Carlo settings
☒	☐	For hierarchical and complex designs, identification of the appropriate level for tests and full reporting of outcomes
☒	☐	Estimates of effect sizes (e.g. Cohen's *d*, Pearson's *r*), indicating how they were calculated

Our web collection on statistics for biologists contains articles on many of the points above.

Software and code

Policy information about availability of computer code

Data collection	Not applicable
Data analysis	All analyses were conducted using R (v 4.3.0). Code-lists for all long-term conditions included in this paper are available for other researchers on https://github.com/rprigge-uoe/mltc-codelists.git.

For manuscripts utilizing custom algorithms or software that are central to the research but not yet described in published literature, software must be made available to editors and reviewers. We strongly encourage code deposition in a community repository (e.g. GitHub). See the Nature Portfolio guidelines for submitting code & software for further information.

Data

Policy information about availability of data

All manuscripts must include a data availability statement. This statement should provide the following information, where applicable:

- Accession codes, unique identifiers, or web links for publicly available datasets
- A description of any restrictions on data availability
- For clinical datasets or third party data, please ensure that the statement adheres to our policy

All bona fide researchers in academic, commercial, and charitable settings can apply to use the UK Biobank resource for health-related research in the public interest (www.ukbiobank.ac.uk/register-apply/). The study was conducted using the UK Biobank Resource under application number 57213. Code-lists for all long-term conditions included in this paper are available for other researchers on https://github.com/rprigge-uoe/mltc-codelists.git.

Human research participants

Policy information about studies involving human research participants and Sex and Gender in Research.

Reporting on sex and gender	Information on the sex of participants was acquired from central registry at recruitment, but in some cases updated by participants. Hence information on sex may contain a mixture of the sex the National Health Service had recorded for the participant and self-reported sex.
Population characteristics	UK Biobank is a national resource that has been described extensively elsewhere (hhttps://www.ukbiobank.ac.uk).
Recruitment	All people aged 40-69 years (men and women) who were registered with the National Health Service living up to 25 miles from one of the 22 study assessment centrs were invited to participate in 2006-2010. Overall, about 9.2 million invitations were mailed out to recruit 503,325 participants (a response rate of 5.5%).
Ethics oversight	Analyses of UKB data are conducted under generic approval from the NHS North West Research Ethics Service (Ref 21/NW/0157) based on individual written informed consent at baseline assessment. Participants who subsequently withdrew consent were excluded from the analysis.

Note that full information on the approval of the study protocol must also be provided in the manuscript.

Field-specific reporting

Please select the one below that is the best fit for your research. If you are not sure, read the appropriate sections before making your selection.

☒ Life sciences ☐ Behavioural & social sciences ☐ Ecological, evolutionary & environmental sciences

For a reference copy of the document with all sections, see nature.com/documents/nr-reporting-summary-flat.pdf

Life sciences study design

All studies must disclose on these points even when the disclosure is negative.

Sample size	172,563 participants
Data exclusions	Our study population included UK Biobank participants aged 40–71 years at baseline, recruited in 22 assessment centres in England, Scotland and Wales from 2006 to 2010, with a continuous primary care record extending from at least a year prior to and up to the day of their baseline assessment. Participants who subsequently withdrew consent were excluded from the analysis.
Replication	N/A
Randomization	N/A
Blinding	N/A

Reporting for specific materials, systems and methods

We require information from authors about some types of materials, experimental systems and methods used in many studies. Here, indicate whether each material, system or method listed is relevant to your study. If you are not sure if a list item applies to your research, read the appropriate section before selecting a response.

Materials & experimental systems

n/a	Involved in the study	
☒	☐	Antibodies
☒	☐	Eukaryotic cell lines
☒	☐	Palaeontology and archaeology
☒	☐	Animals and other organisms
☒	☐	Clinical data
☒	☐	Dual use research of concern

Methods

n/a	Involved in the study	
☒	☐	ChIP-seq
☒	☐	Flow cytometry
☒	☐	MRI-based neuroimaging
